# PTX3 mediates the infiltration, migration, and inflammation‐resolving‐polarization of macrophages in glioblastoma

**DOI:** 10.1111/cns.13913

**Published:** 2022-07-20

**Authors:** Hao Zhang, Yifan Wang, Yihan Zhao, Tao Liu, Zeyu Wang, Nan Zhang, Ziyu Dai, Wantao Wu, Hui Cao, Songshan Feng, Liyang Zhang, Quan Cheng, Zhixiong Liu

**Affiliations:** ^1^ Department of Neurosurgery, Xiangya Hospital Central South University Changsha China; ^2^ National Clinical Research Center for Geriatric Disorders Xiangya Hospital, Central South University Changsha China; ^3^ Xiangya School of Medicine Central South University Changsha China; ^4^ College of Bioinformatics Science and Technology Harbin Medical University Harbin China; ^5^ Department of Oncology, Xiangya Hospital Central South University Changsha China; ^6^ Department of Psychiatry, The Second People's Hospital of Hunan Province The Hospital of Hunan University of Chinese Medicine Changsha China

**Keywords:** cellular communication, glioma microenvironment, macrophage, PTX3, transcription factor

## Abstract

**Introduction:**

Pentraxin 3 (PTX3) is an essential regulator of the immune system. However, the immune‐modulatory role of PTX3 in the tumor microenvironment of glioma has not been elucidated.

**Methods:**

The RNA seq samples were obtained from The Cancer Genome Atlas (TCGA) and the China Glioma Genome Atlas (CGGA) datasets. The single‐cell sequencing data of glioblastoma (GBM) samples were obtained from the Single Cell Portal platform (http://singlecell.broadinstitute.org). Immunohistochemistry was used to assess PTX3 expression, HAVCR2, PD‐1, PD‐L1, and CD276 in glioma sections from the Xiangya cohort (*n* = 60). Multiplex immunofluorescence staining of PTX3, CD68, and CD163 was performed in several solid cancer types, including GBM. HMC3 was cocultured with U251 and U87, and transwell assay and flow cytometry assay were performed to explore the migration and polarization activity of HMC3.

**Results:**

PTX3 expression is significantly increased in GBM. PTX3 expression predicts worse survival in the Xiangya cohort. PTX3 is closely related to the expression of PD‐1, PD‐L1, CD276, and HAVCR2 in the tumor microenvironment. Additionally, PTX3 is involved in tumorigenic and immunogenic processes, especially the activity of macrophages based on various signaling pathways in cellular communications and critical transcription factors. Specifically, PTX3 actively mediates macrophages' infiltration, migration, and inflammation‐resolving‐polarization. PTX3 could also predict immunotherapy response.

**Conclusion:**

PTX3 is critically involved in macrophage infiltration, migration, and inflammation‐resolving‐polarization and modulates an immunosuppressive microenvironment.

## Graphical abstract

In summary, we revealed the characteristics of PTX3 in the development of gliomas and the tumor immune microenvironment of cancer. The immunosuppressive property of PTX3 makes it a potential new therapeutic target and prognostic marker for the treatment of gliomas. Notably, PTX3 was found to mediate the infiltration, migration, and polarization of macrophages in GBM. Our study was expected to provide the background support for further in‐depth research about PTX3 in gliomas.

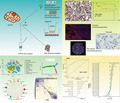

## 1 INTRODUCTION

Glioma, which originates in the neuroglia cells and accounts for about 30% of all tumors in the central nervous system,^1^ is one of the most common and malignant tumors.^2^, ^3^ Recently, primary gliomas are more common in the middle‐aged and elderly population with the increasing incidence date. Since gliomas are characterized by infiltration^4^and invasiveness,^5^ glioma patients have a poor prognosis.

In the tumor microenvironment of gliomas, classic immune checkpoint molecules could act on tumor cells and sometimes lead to the immune escape of tumor cells.^6^, ^7^ Research on the immune checkpoint inhibitors to block the immune escape of tumor cells has demonstrated promising results. Although many drugs targeting immune checkpoints have little effect in extending the survival of glioma patients, immunotherapy is still considered a good therapeutic strategy in gliomas. In particular, the discovery of the intracranial lymphatic system,^8^ anti‐PD‐1 antibody,^9^ and anti‐CTLA‐4 antibody^10^ have shed light on the research about novel drugs.

PTX3 is a soluble inflammatory factor in the tumor microenvironment, a candidate marker of inflammation,^11^ and a congenital immunomodulator associated with immune escape,^12^ which PTX3 has the functions of activating complement, neutralizing pathogens, affecting apoptotic cells, and regulating inflammation.^13^ PTX3 could recruit and combine with Factor H to inhibit tumor‐related inflammation by regulating complement‐dependent tumor‐related inflammation, thus acting as an exogenous tumor suppressor to inhibit tumor‐related inflammation.^14^, ^15^ Moreover, PTX3 acts as a tumor suppressor by blocking the action of pre‐tumor growth factors.^16^, ^17^, ^18^ However, PTX3 has a protective effect against cancer and has been found to correlate with tumor invasion and metastasis in head and neck squamous cell carcinoma, cervical cancer, and other cancers.^19^, ^20^ Notably, down‐regulation of PTX3 leads to the inhibition of tumor growth and development,^15^ while up‐regulation of PTX3 promotes the proliferation and invasion of glioma cells.^21^ PTX3 is also a vital target of the oncogenic phosphoinositide 3‐kinase signaling pathway to promote stem cell‐like traits in basal‐like breast cancers.^22^ PTX3 is a double‐edged sword in carcinogenesis, which depends on the specific tumor microenvironment. Meanwhile, PTX3 has been associated with the tumor grade and malignancy of gliomas.^23^ It should be noted that our previous finding proved that PTX3 promoted GBM progression by negatively regulating cell autophagy.^23^ Taken together, PTX3 has the potential to become a diagnostic marker for gliomas.

However, the immune characteristics and clinical significance of PTX3 have not been adequately elucidated in gliomas. To comprehensively define the role of PTX3 in the tumor microenvironment of gliomas, large‐scale bioinformatics analyses and in vitro experiments were performed. This is the first extensive study of the prognostic value and immune‐related characteristics of PTX3 in gliomas, with particular attention being paid to the potential role of PTX3 in regulating macrophages. It is expected that characterizing the landscape of PTX3 in glioma will help promote the clinical management of glioma.

## 2 MATERIALS AND METHODS

### 2.1 Sample and data collection

We collected LGG and GBM samples from The Cancer Genome Atlas (TCGA) and the China Glioma Genome Atlas (CGGA) datasets. A total of 672 samples from the TCGA dataset were downloaded from UCSC Xena (https://xenabrowser.net/). A total of 1013 samples from CGGA were downloaded from the CGGA website (http://www.cgga.org.cn/). The RNA‐seq data of tumor anatomic structure in GBM was from Ivy Project (http://glioblastoma.alleninstitute.org/). The single‐cell sequencing data of 8953 cells from 33 human primary GBM samples were obtained from the Single Cell Portal platform (http://singlecell.broadinstitute.org) (accession number SCP50 and SCP393) and processed using Smart‐seq2 method. This study was approved by the Ethics Committee of Xiangya Hospital, Central South University. Written informed consent was obtained from all patients.

### 2.2 Immunohistochemistry

Patients (*n* = 60) undergoing the neurosurgical removal of glioma (WHO grade II‐IV) in Xiangya Hospital, Central South University, were the tissue sources (Table S1). Slices (4 μm) from glioma (WHO grade II‐IV) tissues were fixed by formalin and embedded in paraffin. Sections were then boiled with sodium citrate buffer for antigen retrieval, and 3% H_2_O_2_ was used for blocking endogenous peroxidase activity. A 5% BSA was used for section blocking. Rabbit polyclonal anti‐PTX3 (1:50; Proteintech; Wuhan, China), anti‐HAVCR2 (1:500; Proteintech; Wuhan, China), anti‐PD‐1 (1:400; Proteintech; Wuhan, China), anti‐PD‐L1 (1:5000; Proteintech; Wuhan, China), and anti‐CD276 antibody (1:400; Proteintech; Wuhan, China) were used as the primary antibody. Sections were incubated at 4°C overnight. Horseradish peroxidase (HRP) conjugated secondary antibody and 3,3′‐diaminobenzidine were used for visualization. Hematoxylin was used for counterstaining with an optical microscope used for observation. H‐score was calculated by intensity score * quantity score. As for intensity scores, 0, 1, 2, and 3 represented negative, weak, moderate, and strong, respectively. The quantity score was determined by the proportion of stained cells, in which 0, 1, 2, 3, and 4 represented <10%, 10%–25%, 25%–50%, 50%–75%, and >75%, respectively. The H‐score had a range of 0–12.

### 2.3 Genomic alterations

Somatic mutations and copy number variations (CNVs) were downloaded from the TCGA dataset. OncoPrint was used to delineate the somatic mutation landscape of TCGA via the maftools R package.^24^ CNVs associated with PTX3 expression and the threshold copy number at alteration peaks were from GISTIC 2.0 analysis (https://cloud.genepattern.org). We investigated CNVs and the mutation landscape of PTX3 in pan‐cancer from the cBioPortal for cancer genomics (http://www.cbioportal.org), an open‐access repository of cancer genomics datasets.^25^, ^26^

### 2.4 Immune‐related functional annotation

The R package GSVA was applied to analyze the GO terms of the immune‐related process. MCP‐counter and ssGSEA algorithms were used for the quantification of immune cells. Immune infiltration pattern related to PTX3 expression of pan‐cancer samples was explored.

### 2.5 Single‐cell sequencing analysis

Data processing of the GBM single‐cell sequencing samples was performed using the R package “Seurat”. A K‐nearest neighbor was constructed using the “FindNeighbors” function after performing the principal component analysis (PCA) using the “RunPCA” function. The “FindClusters” function was used to integrate cells with the highest gene alteration. The R package “Copykat” was used to annotate aneuploid malignant cells. Non‐malignant cell clusters were identified using the R package “scCATCH.” The “FindMarkers” function screened out the significantly differentially expressed genes (DEGs) between identified microenvironment cells. Neftel has defined four molecular types of GBM at the single‐cell level,^27^ and the corresponding GBM cell subtypes were identified using the “Scalop” algorithm in this study.

Cell–cell interaction analysis was performed using the R package “CellChat.” The specific interaction between malignant cells with high or low PTX3 expression and other identified cell types in different receptor‐ligand signaling pathways was analyzed and visualized.^28^ Gene regulatory network analysis was performed using the R package “SCENIC”^29^ based on regulons and DNA motif co‐expression. The activity of each regulon in the pseudo‐cell was scored using the R package “AUCell.” Regulon modules were identified using the Connection Specificity Index.

### 2.6 Multiplex immunofluorescence staining in GBM samples from the Xiangya cohort

Paraffin sections were deparaffinized. After antigen retrieval, sections were blocked with 3% H_2_O_2_ and 2% BSA. Different primary antibodies, PTX3 (Rabbit, 1:100, AiFang biological, China), CD68 (Mouse, 1:100, AiFang biological, China), and CD163 (Rabbit, 1:3000, Proteintech, China) were sequentially applied, followed by horseradish peroxidase‐conjugated secondary antibody incubation (PV6001, PV6002, ZSGB‐BIO, China) and reaction liquid (CY5‐TYR, CY3‐TYR, and 594‐TYR [RecordBio, China]). After labeling with the human antigens, nuclei were stained with 4′,6‐diamidino2‐phenylindole dihydrochloride (DAPI), and an antifade mounting medium was applied. Stained slides were scanned using the Pannoramic Scanner (3D HISTECH, Hungary) to obtain multispectral images. DAPI glows blue by UV excitation wavelength 330–380 nm and emission wavelength 420 nm in fluorescence spectra. CY3 glows yellow by excitation wavelength 510–560 nm and emission wavelength 590 nm. The 594 glows red by excitation wavelength 594 nm and emission wavelength 615 nm. CY5 glows pink by excitation wavelength 608–648 nm and emission wavelength 672–712 nm. Multispectral images were analyzed, and positive cells were quantified at a single‐cell level by Caseviewer (C.V 2.3 and C.V 2.0) and Pannoramic viewer (P.V 1.15.3) image analysis software.

### 2.7 Multiplex immunofluorescence staining in pan‐cancer samples

The pan‐cancer tissue array was purchased from the Outdo Biotech company (HOrg‐C110PT‐01) and the ethics was approved. Paraffin sections were deparaffinized. After antigen retrieval, sections were blocked with 3% H_2_O_2_ and 2% BSA. Different primary antibodies, PTX3 (Rabbit, 1:100, AiFang biological, China), CD68 (Rabbit, 1:3000, Servicebio, China), and CD163 (Rabbit, 1:3000, Proteintech, China) were sequentially applied, followed by horseradish peroxidase‐conjugated secondary antibody incubation (GB23301, GB23303, Servicebio, China) and tyramide signal amplification (TSA) [FITC‐TSA, CY3‐TSA, and CY5‐TSA (Servicebio, China)]. After labeling with the human antigens, nuclei were stained with DAPI, and an antifade mounting medium was applied. Stained slides were scanned using the Pannoramic Scanner (3D HISTECH, Hungary) to obtain multispectral images. DAPI glows blue by UV excitation wavelength 330–380 nm and emission wavelength 420 nm in fluorescence spectra. CY3 glows yellow by excitation wavelength 510–560 nm and emission wavelength 590 nm. CY5 glows pink by excitation wavelength 608–648 nm and emission wavelength 672–712 nm. FITC glows green by excitation wavelength 465–495 nm and emission wavelength 515–555 nm. Multispectral images were analyzed, and positive cells were quantified at a single‐cell level by Caseviewer (C.V 2.3 and C.V 2.0) and Pannoramic viewer (P.V 1.15.3) image analysis software.

### 2.8 Cell Culture and Transfection

Human GBM cells (U251 and U87) were maintained in DMEM medium with 10% fetal bovine serum (10% DMEM), and microglia cells (HMC3) were maintained in 1640 medium with 10% fetal bovine serum (10% 1640) under 37°C and 5% CO_2_. U251 and U87 cells were randomly divided into the siRNA‐NC (si‐NC) and the siRNA‐PTX3 (si‐PTX3) groups. The siRNA of PTX3 (5′‐GGTCAAAGCCACAGATGTA‐3′) was obtained from RiboBio (Guangzhou, China). A 10 μl siRNA, 12 ul transfection reagent, and 120 μl 1x loading buffer were added to 1858 ul 10% DMEM for the 2 ml transfection system.

### 2.9 Western blotting assay

The western blotting assay assessed PTX3 expression and β‐actin. Anti‐PTX3 (Rabbit, 1:1000, Proteintech, China) and anti‐β‐actin (Mouse, 1:5000, Proteintech, China) were used as the primary antibody. HRP goat anti‐mouse IgG (Mouse, 1:5000, Proteintech, China) and HRP goat anti‐rabbit IgG (Rabbit, 1:6000, Proteintech, China) were used as the secondary antibody. ECL development was used for visualization.

### 2.10 RT‐qPCR assay

The primers of β‐actin (F ACCCTGAAGTACCCCATCGAG; R AGCACAGCCTGGATAGCAAC) and PTX3 (F TGCGATTCTGTTTTGTGCTCT; R GAGCTTGTCCCATTCCGAGT) were designed using the primer 5.0. Total RNAs were extracted and reversely transcribed into cDNA by HiScript Q RT SuperMix for RT‐qPCR. The expression levels of β‐actin and PTX3 were quantified using 2^−ΔΔCT^.

### 2.11 Coculture of HMC3 and U251/U87 cells for transwell assay

U251/U87 cells were cultured in a 6‐well plate at the density of 2 × 10^5^/ml. After being transfected with si‐NC and si‐PTX3, U251/U87 cells were cultured for 24 h. Subsequently, U251/U87 cells were digested and resuspended using 10% DMEM at the density of 2 × 10^5^ each well and were added to the lower chamber. HMC3 cells were also digested and resuspended at the density of 2 × 10^6^/ml, and a 100 μl suspension of HMC3 cells was added to the upper chamber. U251/U87 cells and HMC3 cells were cocultured for 72 h. The upper chamber was washed twice with phosphate buffer saline (PBS) at 24, 48, and 72 h, respectively; the upper chamber was washed twice with PBS. The wet swab was used to wipe the cells on the upper layer. The upper chamber was then fixed using acetone and methyl alcohol at the ratio of 1:1 for 20 min. After being washed with PBS twice, the upper chamber was stained with 0.5% crystal violet for 5 min for photographing.

### 2.12 Coculture of HMC3 and U251/U87 cells for flow cytometry assay

HMC3 cells were also digested and resuspended at the density of 5 × 10^5^ each well and were added to the lower chamber. U251/U87 cells were cultured in a 6‐well plate at the density of 2 × 10^5^/ml. After being transfected with si‐NC and si‐PTX3, U251/U87 cells were cultured for 24 h. Subsequently, U251/U87 cells were digested and resuspended using 10% DMEM at the density of 5 × 10^6^/ml, and 100 μl suspension of U251/U87 cells were added to the upper chamber. U251/U87 cells and HMC3 cells were cocultured for 48 h. HMC3 cells from the lower chamber were digested, and 1 × 10^6^/100 ul cells were added to the 1.5 ml tubes. The suspension was centrifuged at 2000 rpm and washed with PBS twice. The supernatant was discarded, and antibodies of CD68 (Invitrogen, 12‐0689‐41) and CD163 (Invitrogen, 17‐1639‐41/25‐1639‐41) for flow cytometry were added, followed by staining for 30 min at room temperature and in a dark place. CD68 and CD163 in different groups were measured by flow cytometry (Beckman, A00‐1‐1102).

### 2.13 Statistical analysis

Spearman or Pearson correlation analysis was used to evaluate the correlations between continuous variables. Kaplan–Meier survival curves depicted the survival probability. The student *t*‐test was used for normally distributed variables between two groups, while the Wilcoxon test was used for nonnormally distributed variables between two groups. The parametric one‐way analysis of variance and nonparametric Kruskal–Wallis test were used for normally and nonnormally distributed variables among multiple groups. All statistical analyses were performed using the R project (version 3.6.3, https://www.r‐project.org/) and Graphpad Prism 8 (version 8.4.3). *p*‐values <0.05 were considered to be statistically significant. And all tests were two‐sided. Shapiro–Wilk test was used to assess the normal data distribution. All experiments were repeated three times.

## 3 RESULT

### 3.1 Inter‐tumor and intra‐tumor heterogeneous characteristics of PTX3 in gliomas

**FIGURE 1:**
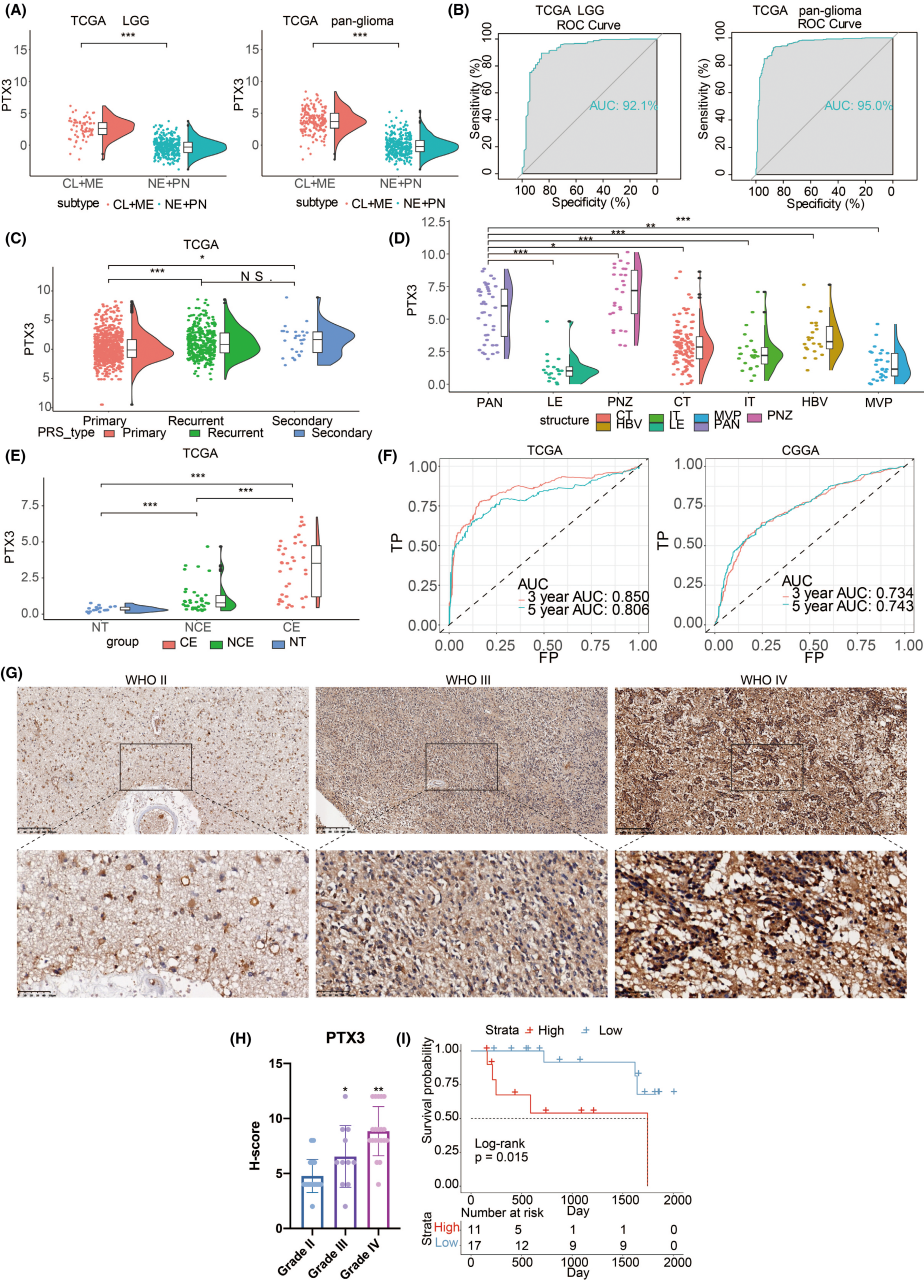
Inter‐tumor and intra‐tumor heterogeneous expression characteristics of PTX3 in gliomas. (A) PTX3 expression in proneural, neural, classical, and mesenchymal subtypes in the TCGA dataset. (B) The ROC curve indicates the sensitivity and specificity of PTX3 expression in predicting the ME and CL subtypes. (C) PTX3 expression in primary, secondary, and recurrent gliomas in the TCGA dataset. (D) Intra‐tumor distribution of PTX3 in LE (Leading Edge), IT (Infiltrating Tumor), CT (Cellular Tumor), PAN (Pseudopalisading Cells Around Necrosis), PNZ (Perinecrotic Zone), MVP (Microvascular Proliferation), and HBV (Hyperplastic Blood Vessels) regions based on Ivy GBM RNA‐seq data. (E) PTX3 expression in different radiographical regions in the TCGA dataset. CE, contrast‐enhanced; NCE, non‐contrast‐enhanced; NT, normal tissue. (F) The ROC curves indicate the sensitivity and specificity of PTX3 in predicting 3‐year and 5‐year survival in the TCGA and CGGA datasets. (G) Representative images of IHC staining for PTX3 in different pathological grades of gliomas in Xiangya cohort. (H) Statistical analysis of H‐score regarding PTX3 expression in Xiangya cohort. (I) Kaplan–Meier analysis of OS of glioma patients based on high vs. low expression of PTX3 (H‐score) in Xiangya cohort.

Human gliomas are divided into four different molecular subclasses: classic (CL), mesenchymal (ME), pro‐neural (PN), and neural (NE), in which CL and ME subtypes show more aggressive behavior than NE or PN subtypes.^30^ PTX3 expression in CL and ME subtypes was significantly higher than NE and PN subtypes in LGG and pan‐glioma from the TCGA dataset (Figure 1A). The area under the receiver operating characteristic curve (AUC) values of PTX3 in predicting CL and ME subtypes were 92.1% and 95.0% in LGG and pan‐glioma from the TCGA dataset, respectively (Figure 1B). These findings indicated that PTX3 might be a biological predictor of CL and ME subtypes. PTX3 had relatively higher expression in recurrent and secondary gliomas than primary gliomas (Figure 1C).

The intra‐tumor distribution of PTX3 in GBM tissues was evaluated. Compared with other pathological regions, PTX3 expression was considerably higher in the Pseudopalisading Cells Around Necrosis (PAN) and Perinecrotic Zone (PNZ) (Figure 1D). In addition, radiologically, there was a significant difference between the contrast‐enhanced and non‐contrast‐enhanced regions regarding PTX3 expression (Figure 1E).

### 3.2 PTX3 expression level is up‐regulated in the order of tumor grade

We further investigated the mRNA expression levels of PTX3 in different grades of gliomas in the CGGA cohort. We found that PTX3 expression was higher in WHO III than in WHO II and higher in WHO IV than in WHO III (*p* < 0.001, Figure S1A). Isocitrate dehydrogenase mutation is a classical mutagenized site in glioma development. PTX3 expression was higher in IDH wildtype than the IDH mutant in the TCGA and CGGA datasets (Figure S1B). The receiver operating characteristic curve (ROC) analysis further indicated that PTX3 expression could predict IDH mutation in LGG and pan‐glioma samples in the CGGA dataset (the AUC value = 0.756, 0.656, respectively; Figure S1C). Besides, the ROC curve indicated that PTX3 expression could predict IDH mutation in LGG and pan‐glioma samples in the TCGA dataset (the AUC value = 0.901 and 0.824, respectively; Figure S1D). Additionally, in glioma samples from the TCGA and CGGA datasets, PTX3 expression was generally up‐regulated in patients with 1p/19q non‐codeletion (Figure S1E,F). Although PTX3 was abnormally up‐regulated in patients with 1p/19q codeletion in LGG samples from the TCGA dataset, this could be partly explained by the insufficient sample size of the TCGA dataset. We next confirmed that the MGMT methylation was associated with PTX3 expression. PTX3 expression was up‐regulated in the MGMT unmethylated LGG samples in the TCGA dataset, while the expression difference was not significant in GBM samples ( Figure S1G). Moreover, the expression pattern of PTX3 regarding histology of gliomas was shown in Figure S1H.

### 3.3 PTX3 expression is associated with poor survival in glioma patients

The prognostic value of PTX3 was further explored based on the TCGA and CGGA datasets. The AUC values for 3‐ and 5‐year survival of glioma patients were 0.850 and 0.806 in TCGA and 0.734 and 0.743 in CGGA, respectively (Figure 1F). IHC staining further showed that PTX3 expression in glioma positively correlated with the malignant degree of gliomas (Figure 1G,H). Glioma samples from the Xiangya cohort were divided into two groups based on the H‐scores of PTX3. Among the cases in our hospital, patients with high PTX3 expression (H‐score >6) had a lower survival probability (Figure 1I). This meant that PTX3 could serve as a predictor for determining the severity and survival rate of patients with gliomas. High expression of PTX3 was associated with reduced progression‐free interval (PFI) and disease‐specific survival (DSS) in LGG and pan‐glioma samples in the TCGA dataset (Figure S2A,B). PTX3 was also associated with reduced overall survival (OS) in LGG, pan‐glioma samples in the CGGA dataset (Figure S2C). These results suggested that PTX3 might be one of the prognostic indicators of gliomas. PTX3 was a hazardous prognostic marker in 18 independent tumor cohorts and a favorable prognostic marker in three independent tumor cohorts regarding OS (Figure S2D). Similar results were observed in the analysis regarding DSS (Figure S2E). The Kaplan–Meier curve also proved that the high expression of PTX3 had poor prognostic significance in 14 other cancers in addition to glioma (Figures S3 and S4). These results revealed that PTX3 might serve as a biomarker to predict the poor prognosis in pan‐cancer.

To verify the independent prognostic significance of PTX3, univariate and multivariate cox regression analyses were performed (Table S2). We found that PTX3, age at diagnosis, WHO grade, 1p19q status, and IDH status were significant independent factors for OS, while gender was irrelevant. Based on the five independent prognostic factors (PTX3 expression, age, 1p19q status, IDH status, and the grade of gliomas), we built a predictive model by nomogram in the TCGA dataset (Figure S5A). Calibration curves of 1‐year OS, 3‐year OS, 4‐year OS, and 5‐year OS in the TCGA dataset (Figure S5B) and a calibration curve of 4‐year OS in the CGGA dataset (Figure S5E) showed that the nomogram did well in predicting patient survival. A high nomogram‐derived score indicated less survival time in the TCGA (Figure S5C) and CGGA (Figure S5F) datasets. The ROC analysis further noted that the nomogram‐derived score was an ideal marker for predicting the survival time of the glioma patients in the TCGA and CGGA datasets (Figure S5D, AUC = 0.878; Figure S5G, AUC = 0.803).

### 3.4 PTX3 expression levels are associated with distinct genomic alterations

To determine whether the level of PTX3 expression was associated with specific genomic characteristics in gliomas, we performed CNV and somatic mutation analysis in the TCGA dataset. The overall CNV profile was obtained (Figure S6A). The deletion of 1p36, 9p21, and the amplification of 1p32, 7p11, and 12q13‐15 were more related to glioma with high expression of PTX3. The deletions of 4q, 9p, 13q, 19q, and the amplification of 4q12, 8q23‐24, 12p13‐14, and 19q more regularly occurred in gliomas with low expression PTX3 (Figure S6B). Analysis of somatic mutation profiles based on PTX3 expression levels showed that IDH1 (89%), TP53 (59%), and ATRX (42%) were more frequently mutated in the PTX3 low group, and EGFR (29%), PTEN (24%), and TTN (24%) were more commonly mutated in the PTX3 high group (Figure S6C). We then employed cBioPortal to inspect the mutation frequency of PTX3 in the TCGA dataset, and we found that LUSC (exceeding 20%), ESCA (exceeding 14%), and CESC (exceeding 10%) shared relatively high mutation levels with the PTX3 alteration frequency (Figure S6D,E). We also analyzed the relationship between copy number (CN) and PTX3 expression levels (Figure S7A–C). In pan‐gliomas, tumors with CN loss had lower expression of PTX3 compared to tumors with CN gain. These results demonstrated that PTX3 expression was associated with chromosomal changes in human gliomas.

### 3.5 PTX3 is associated with immunomodulatory and inflammatory activities in gliomas

To identify the PTX3‐associated immune functions in glioma, we performed Gene Ontology (GO) analysis in the TCGA dataset (Figure S7D,E) and CGGA dataset (Figure S7F,G). The result showed that differentiation of Th1, Th2, Treg cells, Th1 immune response, and fibroblasts proliferation positively correlated with PTX3 expression. The increased PTX3 expression was also accompanied by the negative regulation of activation and differentiation of B cells and CD4+T cells and the negative regulation of proliferation of T cells. These results suggested that PTX3 could potentially play critical dual roles in immune activation and immune suppression.

**FIGURE 2:**
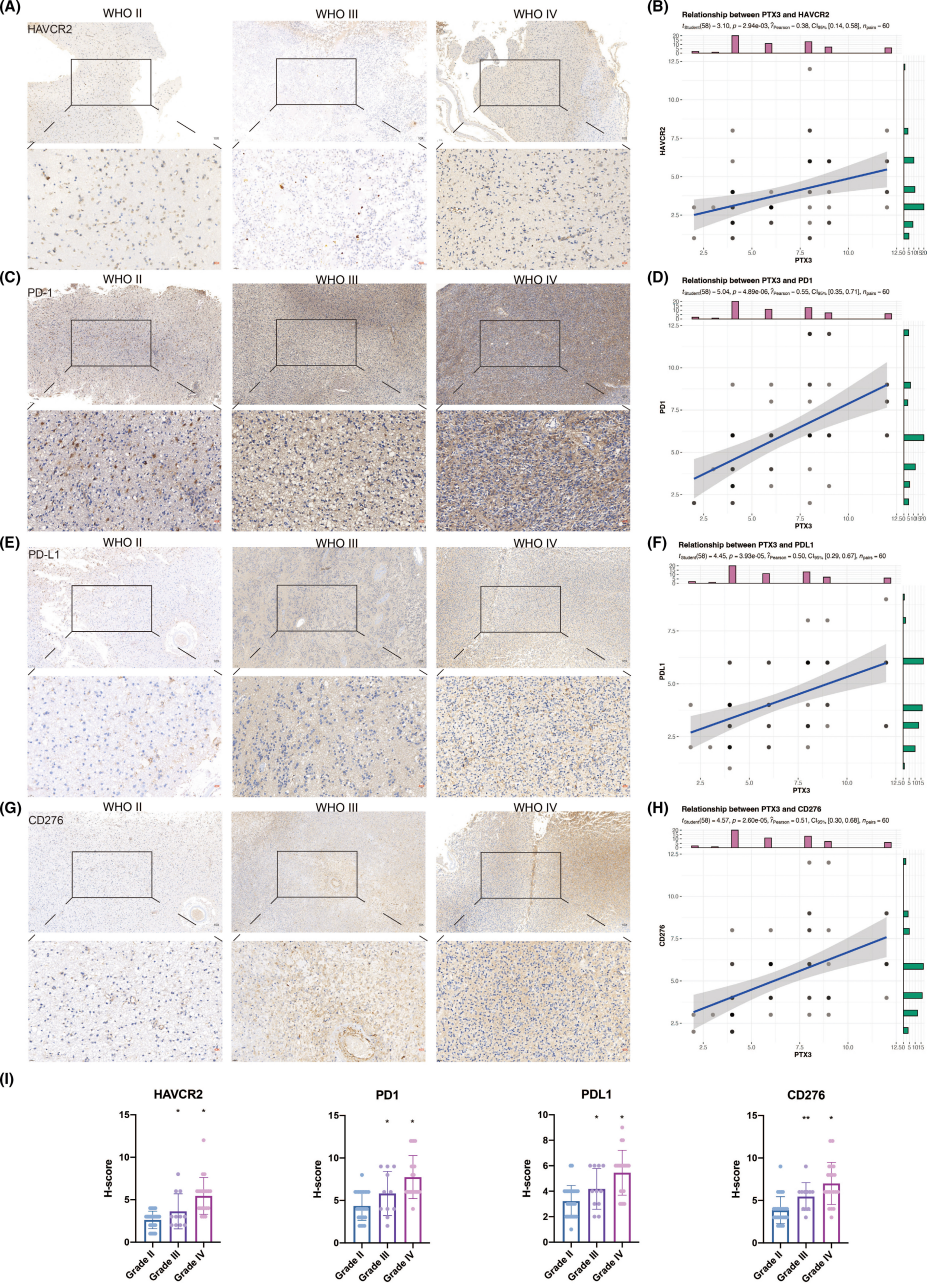
IHC staining for classical immune checkpoints. (A) Representative images of IHC staining for HAVCR2 in different pathological grades of gliomas. (B) Scattering plot depicting the correlation between HAVCR2 and PTX3 based on the H‐score. (C) Representative images of IHC staining for PD‐1 in different pathological grades of gliomas. (D) Scattering plot depicting the correlation between PD‐1and PTX3 based on the H‐score. (E) Representative images of IHC staining for PD‐L1 in different pathological grades of gliomas. (F) Scattering plot depicting the correlation between PD‐L1and PTX3 based on the H‐score. (G) Representative images of IHC staining for CD276 in different pathological grades of gliomas. (H) Scattering plot depicting the correlation between CD276 and PTX3 based on the H‐score. (I) Statistical analysis of H‐score regarding HAVCR2, PD‐1, PD‐L1, and CD276 expression in Xiangya cohort.

The correlation between PTX3 and various inflammation‐related signatures was explored. PTX3 expression positively correlated with MHC_1, MHC_II, HCK, LCK, interferon, and STAT1 and negatively correlated with lgG (Figure S8A–D). Given the vital functions of immune checkpoint molecules in the regulation of immune processes, we analyzed the correlation between PTX3 and classic immune checkpoint molecules. Based on the TCGA and CGGA datasets, we proved that PTX3 positively correlated with CD276, CD274, PDCDL1LG2, HAVCR2, CD80, IDO1, and PDCD1 in pan‐glioma, GBM, and LGG (Figure S8E,F). In the Xiangya cohort, the IHC staining of HAVCR2, PD‐1, PD‐L1, and CD276 was also performed. These four classical immune checkpoint molecules had increased expression in line with gliomas' high tumor grade (Figure 2A,C,E,G). Based on the H‐scores, the correlation between PTX3 and HAVCR2, PD‐1, PD‐L1, and CD276 was calculated, respectively (Figure 2I). The correlation coefficient between PTX3 and HAVCR2 was 0.38 (Figure 2B). The correlation coefficient between PTX3 and PD‐1 was 0.55 (Figure 2D). The correlation coefficient between PTX3 and PD‐L1 was 0.50 (Figure 2F). The correlation coefficient between PTX3 and CD276 was 0.51 (Figure 2H). The above results confirmed the close relationship between PTX3 and immune checkpoint molecules.

We also found that PTX3 expression was significantly positively related to immune cells (Figure S9A), including monocytes, neutrophils, T cells, B cells, NK cells, fibroblasts, and endothelial cells (Figure S10). Further, in most cancers, PTX3 was involved in multiple immune regulation‐related biological pathways, such as the activation and proliferation of fibroblasts, and the immune defense and cytotoxicity of T cells and macrophages (Figure S9B). These results suggested that PTX3 could participate in regulating a variety of immune infiltrating cells.

### 3.6 Molecular characteristics of PTX3 at single‐cell sequencing level

**FIGURE 3:**
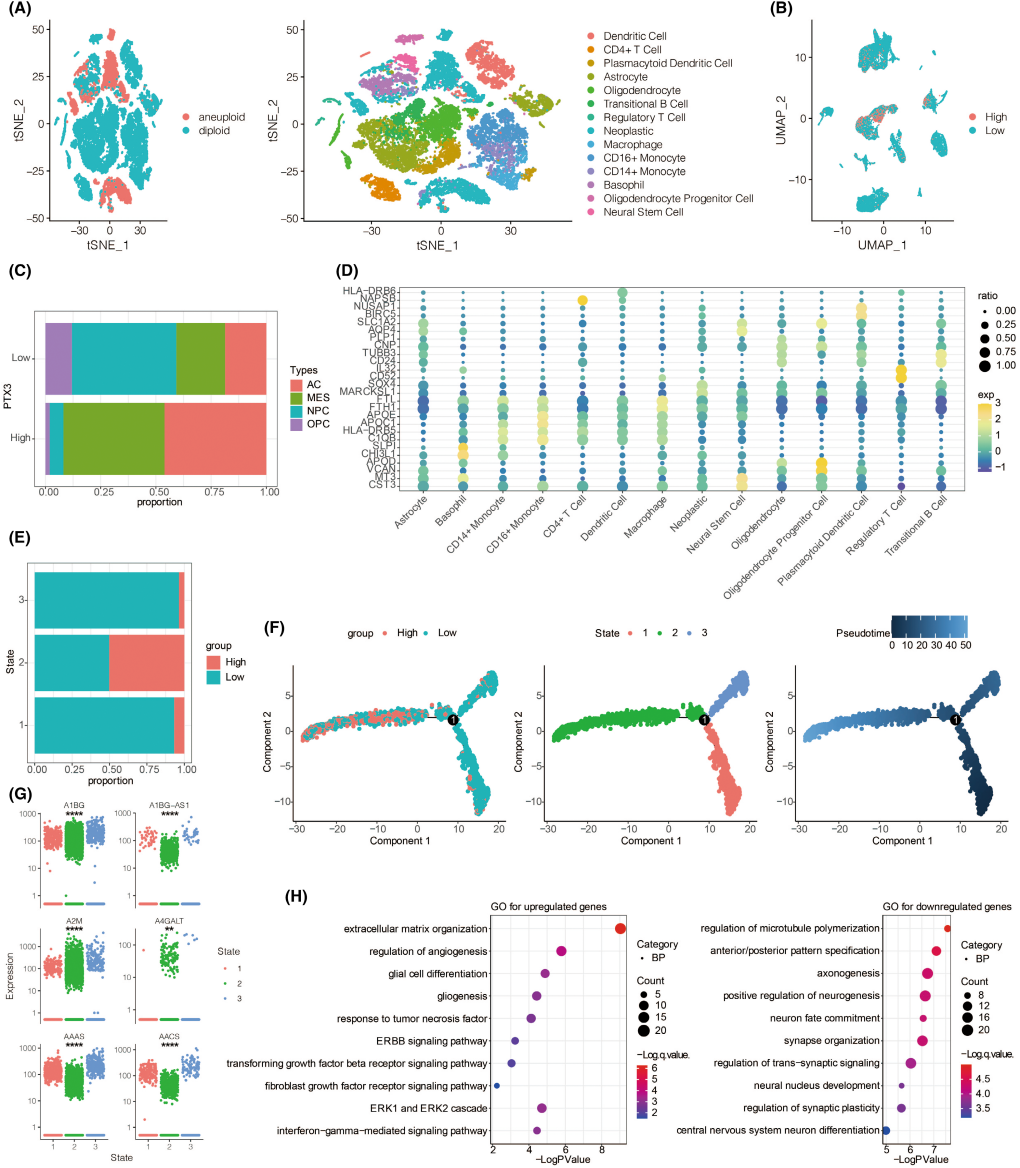
Molecular features of PTX3 at the single‐cell level. (A) t‐SNE for the dimension reduction and visualization of aneuploid cells, diploid cells, and other 14 cell types within the tumor microenvironment. (B) UMAP for the dimension reduction and visualization of cells with high or low PTX3 expression. (C) Relative proportion of four cell types in cells with high or low PTX3 expression. (D) The differentially expressed genes among the identified 14 cell types. E. PTX3 expression in three cell states based on pseudotime analysis. (F) Pseudotime trajectory analysis based on PTX3 expression. (G) Top six differentially expressed genes between high and low PTX3 expression. (H) GO and KEGG enrichment analysis of differentially expressed genes between high and low PTX3 expression.

We next explored the characteristics of PTX3 in 33 GBM samples based on single‐cell sequencing analysis. R package Copykat identified aneuploid cells and diploid cells, and aneuploid cells were defined as neoplastic cells (Figure 3A). A total of 14 cell types were further identified (Figure 3A). PTX3 expression was shown in Figure 3B. GBM cells have four main cell types at single‐cell sequencing levels: neural‐progenitor‐like (NPC‐like), oligodendrocyte‐progenitor progenitor‐like (OPC‐like), astrocyte‐like (AC‐like), and mesenchymal‐like (MES‐like) based on the expression spectrum of GBM cells elucidated by Neftel.^27^ We found that AC‐like and MES‐like state malignant cells were associated with high PTX3 expression, whereas OPC‐like and NPC‐like state malignant cells exhibited low PTX3 expression (Figure 3C). Malignant cells with high PTX3 expression exhibited greater immunogenic and tumorigenic pathways (Figure S11A). The DEGs among the 14 cell types were visualized in Figure 3D. Pseudotime trajectory analysis was performed using monocle and defined three cell states based on one branch point. Cells would gradually differentiate from cell state 1 to cell states 2 and 3 as pseudotime increased (Figure 3F). Notably, PTX expression increased as pseudotime increased (Figure 3F) and was most expressed in state 2 compared with states 1 and 3 (Figure 3E). The top six DEGs among the three cell states are shown in Figure 3G. The top 100 downregulated and upregulated genes with the increase in pseudotime are shown in Figure S11B. GO enrichment analysis revealed that as pseudotime increased, upregulated genes were enriched in tumorigenic processes while downregulated genes were enriched in synapse regulation (Figure 3H).

### 3.7 Cellular interaction and transcription factor related to PTX3

**FIGURE 4:**
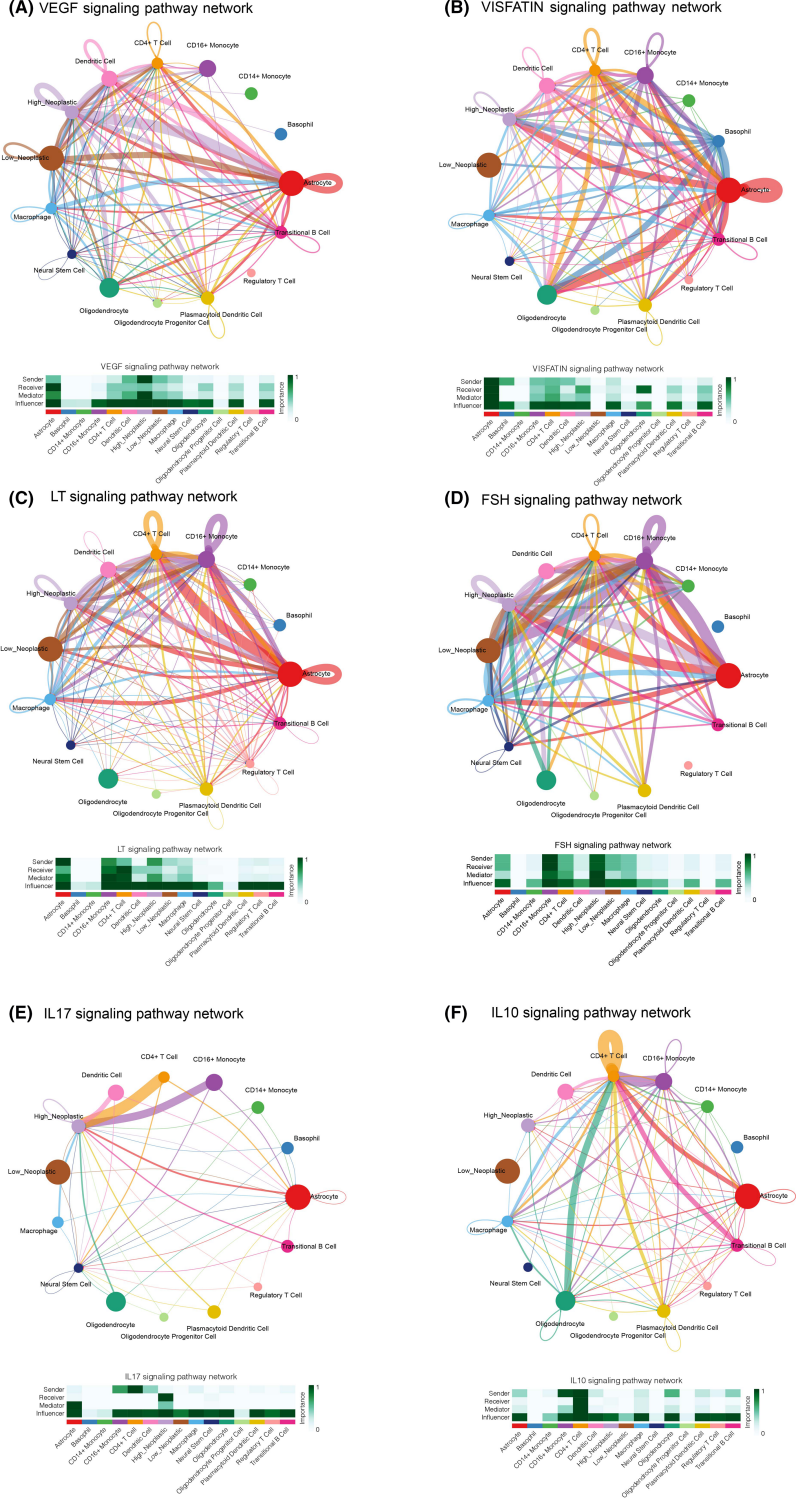
Cellular interaction within the two neoplastic cell clusters with different PTX3 expressions. The cellular interaction network identified cell clusters in various signaling pathways, including A. VEGF, B. VISFATIN, C. LT, D. FSH, E. IL17, and F. IL10 signaling pathways.

The cellular communication of neoplastic cells with high and low PTX3 expression was explored. Overall, the roles of the 15 cell types in cellular communication were classified into receiver, sender, mediator, and influencer. The cellular communication patterns of the receiver and sender of the identified 15 cell types were divided into three distinct types using the “CellChat” R package (Figure S11C,E, respectively). The specific genes involved in the receiver and sender communication pattern of the 15 immune cell types also exhibited three distinct patterns (Figure S11D,F, respectively). The dot plot depicted the receiver and sender communication (Figure S12A) patterns of 15 cell types. Sankey plot revealed that astrocyte, neural stem cell, high neoplastic cell, and low neoplastic cell correlated with signaling pathways of the receiver in pattern 1, while astrocyte, basophil, neural stem cell, high neoplastic cell, and low neoplastic cell correlated with signaling pathways of the sender in pattern 1 (Figure S12B, respectively). The association between PTX3 expression and specific signaling pathways was further elucidated. In general, GBM cells with high expression of PTX3 showed a strong interaction with microenvironment cells via the ADIPONECTIN, FASLG, ANGPT, GH, GRN, IL6, IFN‐I, IFN‐II, PERIOSTIN, PROS, TWEAK, SEMA3, and SPP1 signaling pathways (Figures S13–S15). GBM cells with low expression of PTX3 showed a strong interaction with microenvironment cells via the EPO, MIF, MK, PSAP, and PTN signaling pathways (Figure S13–S15). Notably, GBM cells with high expression of PTX3 showed strong interaction with immune cells via the VEGF, VISFATIN, LT, FSH, IL17, and IL10 signaling pathways (Figure 4).

**FIGURE 5:**
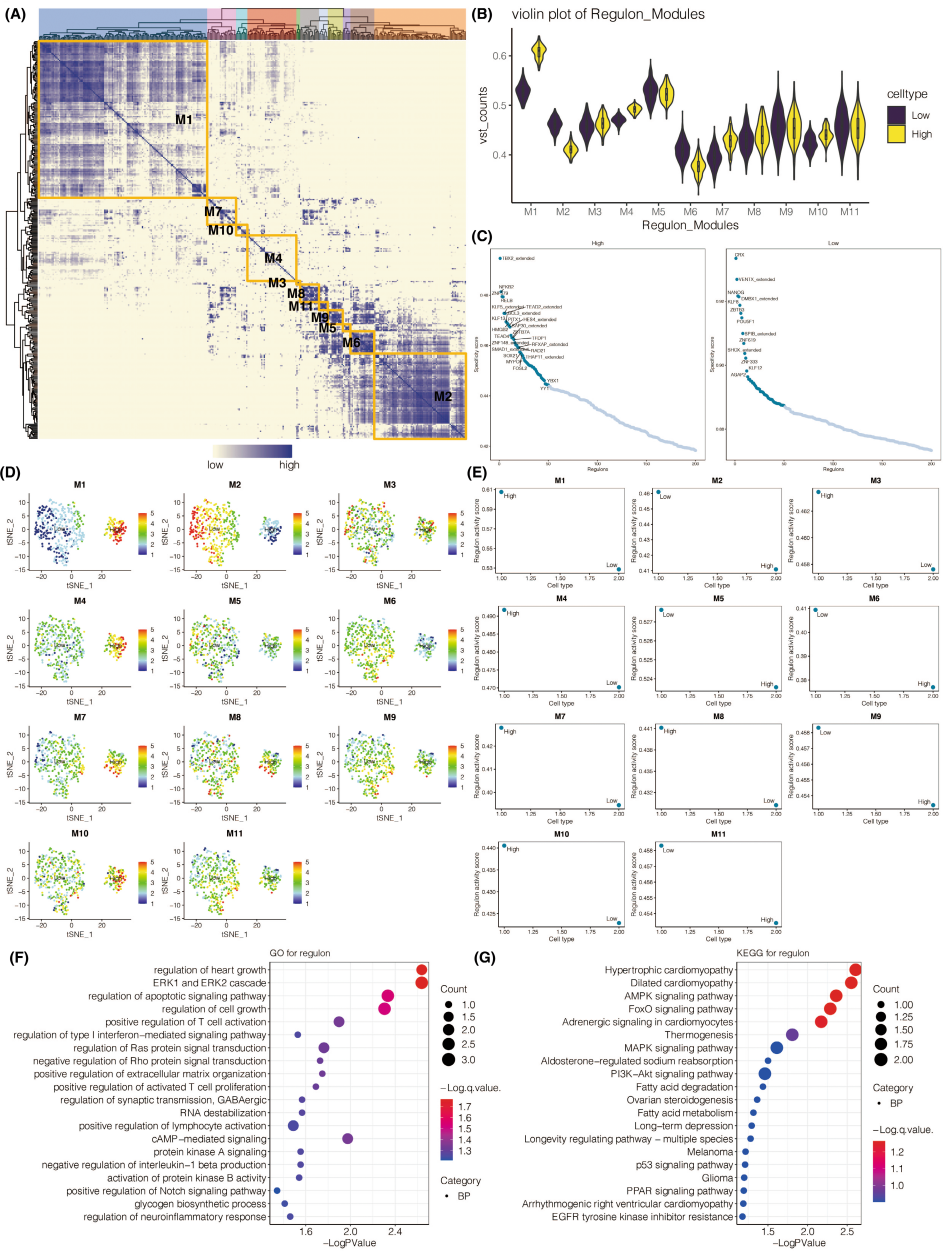
The relationship between PTX3 expression and transcription factors. (A) The heatmap for the distribution of eleven modules of transcription factors in identified malignant cells. B. Violin plot for the regulon levels in malignant cells with high or low PTX3 expression in each regulon module. C. Scattering plot for the distribution of transcription factors in malignant cells based on PTX3 expression. D. t‐SNE plot for the dimension reduction of regulon modules. E. The different regulon levels of malignant cells with high or low PTX3 expression in each module. F. GO enrichment analysis of top‐ranked regulons. G. KEGG enrichment analysis of top‐ranked regulons

It is noteworthy that multiple transcription factors play a critical role in the biological functions of cells within the tumor microenvironment.^31^ We comprehensively analyzed the regulons involved in the complex interaction of 15 cell types. The connection specificity index of each regulon was applied to define the connection between different regulons. Regulons with high index exhibit the ability to co‐regulate downstream genes and might jointly be responsible for the biological functions of cells within the tumor microenvironment. The regulatory subclasses were clustered into 11 main modules based on the calculated connection specificity index, which regulons were more enriched in modules one and two (Figure 5A). Neoplastic cells with high PTX3 expression had more regulons in modules one, seven, and ten (Figure 5B). The key regulons in neoplastic cells with high PTX3 expression were NFKB2, ZNF579, RELB, and KLF5_extended, while key regulons in neoplastic cells with low PTX3 expression included CRX, VENTX_extended, and NANOG (Figure 5C). The t‐SNE plot depicted the dimension reduction of regulon modules (Figure 5D). The different distribution of regulons in neoplastic cells with high or low PTX3 expression in each module is shown in Figure 5E. GO (Figure 5F) and KEGG (Figure 5G) enrichment analysis revealed that the top‐ranked regulons were involved in tumorigenic processes, including regulation of apoptotic signaling pathway and positive regulation of notch AMPK signaling pathway, PI3K‐Akt signaling pathway, p53 signaling pathway.

### 3.8 Multiplex immunofluorescence staining of PTX3, CD68, and CD163

**FIGURE 6:**
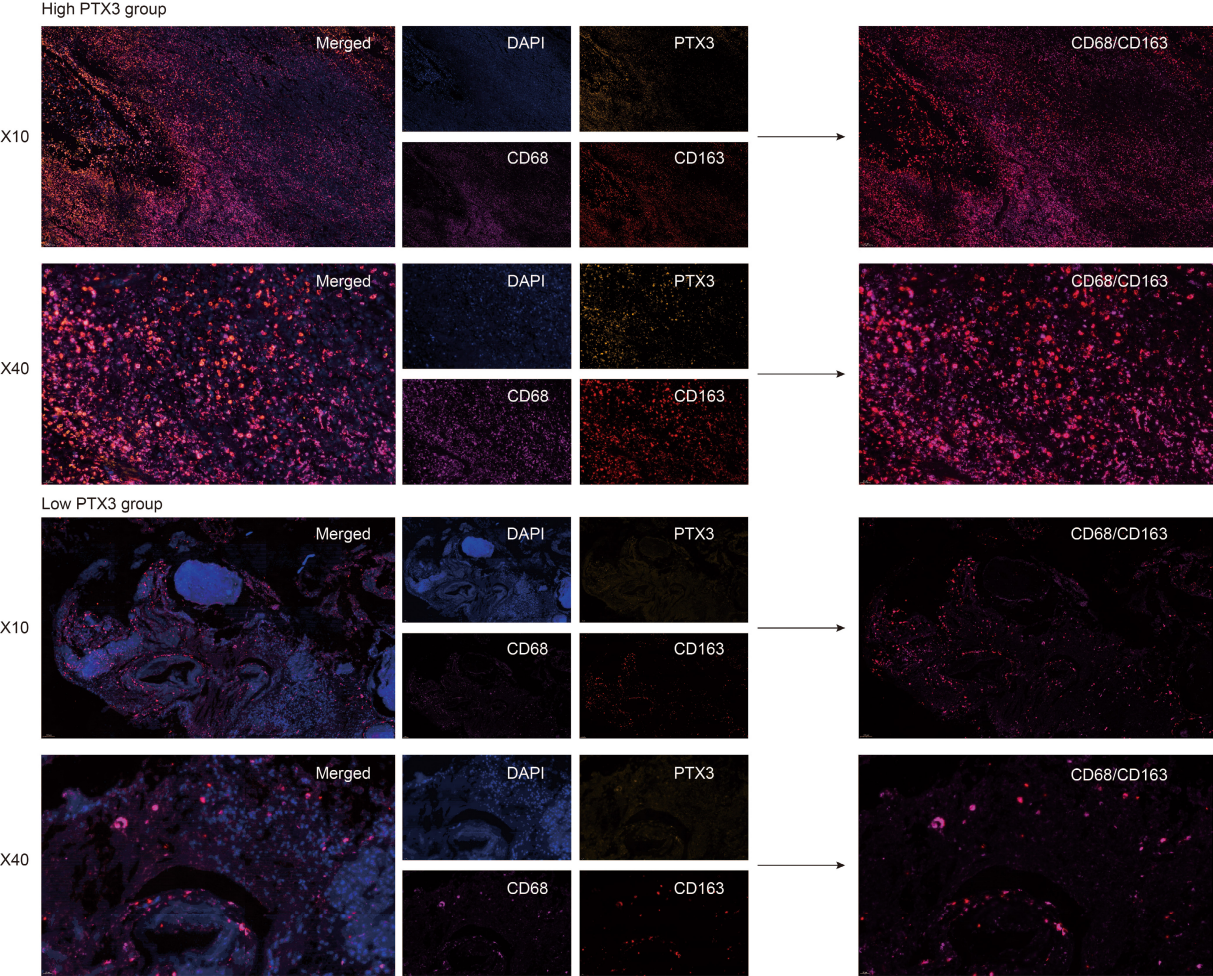
Multiplex immunofluorescence staining of CD68, CD163, PTX3, and DAPI. Multiplex immunofluorescence staining of CD68 (pink), CD163 (red), PTX3 (yellow), and DAPI (blue) in GBM samples from Xiangya cohort (10X and 40X), scale bar 100 and 20 um, respectively.

To prove the potential connection between PTX3 and macrophages, we performed the multiplex immunofluorescence staining of PTX3, CD68, and CD163 in GBM samples from the Xiangya cohort. Notably, in GBM samples with high PTX3 expression, CD68 and CD163 were also abundantly expressed (Figure 6). Further, the co‐expression of CD68 and CD163 was also higher in the high PTX3 group. Moreover, the multiplex immunofluorescence staining of PTX3, CD68, and CD163 was performed in pan‐cancer samples, including astrocytoma, laryngeal squamous cell carcinoma (HNSCC), ureteral urothelial carcinoma, thyroid carcinoma (THCA), urothelial carcinoma of the renal pelvis, bladder carcinoma (BLCA), cervical squamous cell carcinoma and endocervical adenocarcinoma (CESC), uterine corpus endometrial carcinoma (UCEC), squamous cell carcinoma of the penis, ovarian papillary cystadenocarcinoma, serous ovarian adenocarcinoma, testicular germ cell tumors (TGCT), and prostate adenocarcinoma (PRAD) and some corresponding paratumor tissues (Figure S16). Consistently, PTX3 was more co‐expressed with CD68 and CD163 in tumor tissues than in paratumor tissues. Besides, CD68 and CD163 were more co‐expressed in tumor tissues with high PTX3 expression than in tumor tissues with low PTX3 expression.

### 3.9 PTX3 mediates the migration and polarization of microglia

**FIGURE 7:**
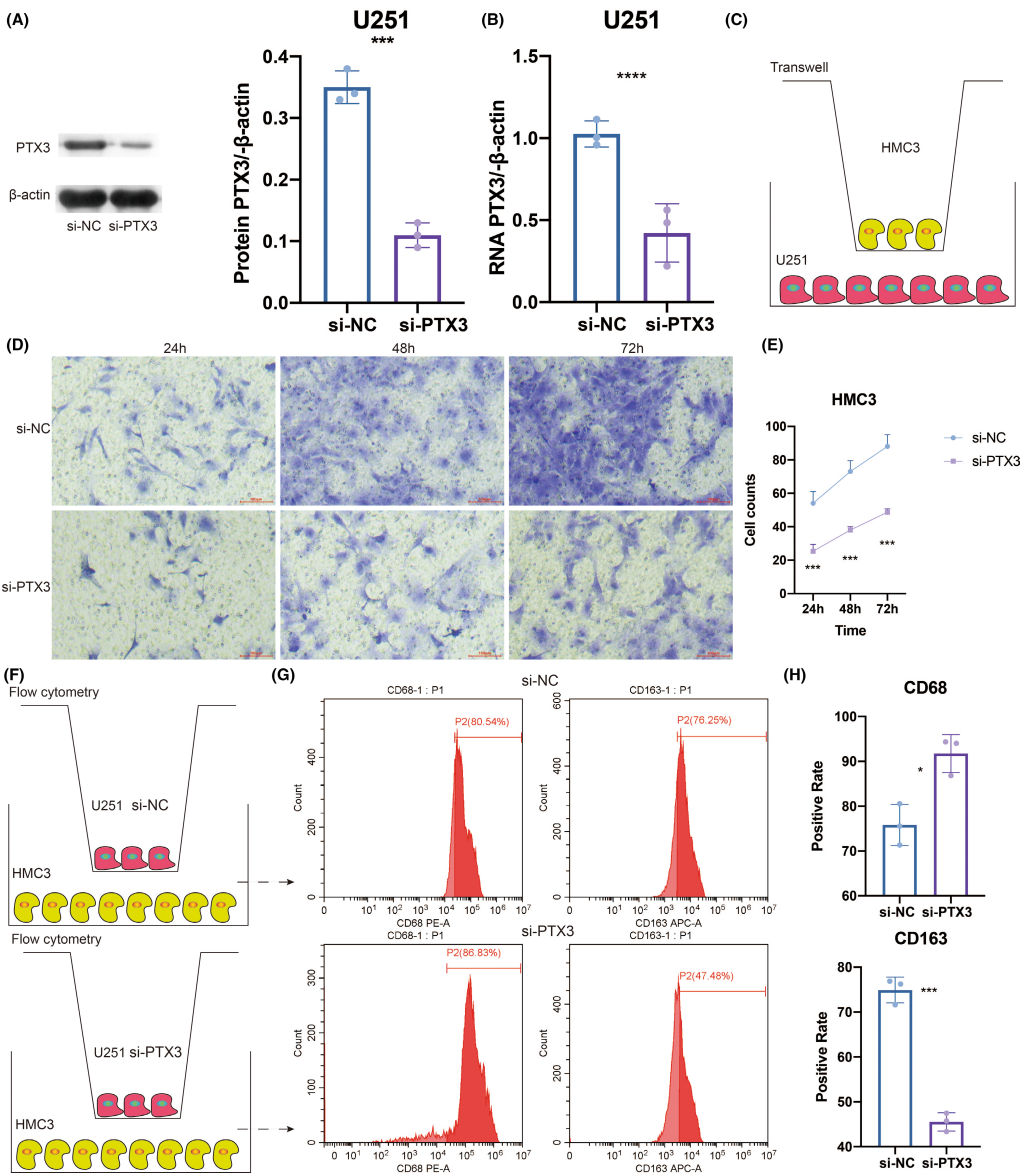
U251‐derived PTX3 mediated the migration and polarization of HMC3. (A) Western blotting results of PTX3 expression in NC and siRNA groups. (B) qPCR results of PTX3 expression in NC and siRNA groups. (C) Study design of the coculture system between HMC3 and U251 cells for transwell assay. (D) Representative images of transwell assay for migration of HMC3 in NC and siRNA groups in different time points. (E) Statistical analysis of transwell assay. (F) Study design of the coculture system between HMC3 and U251 cells for flow cytometry assay. (G) Flow cytometry assay results of CD68 and CD163 expression in NC and siRNA groups. (H) Statistical analysis of flow cytometry assay.

**FIGURE 8:**
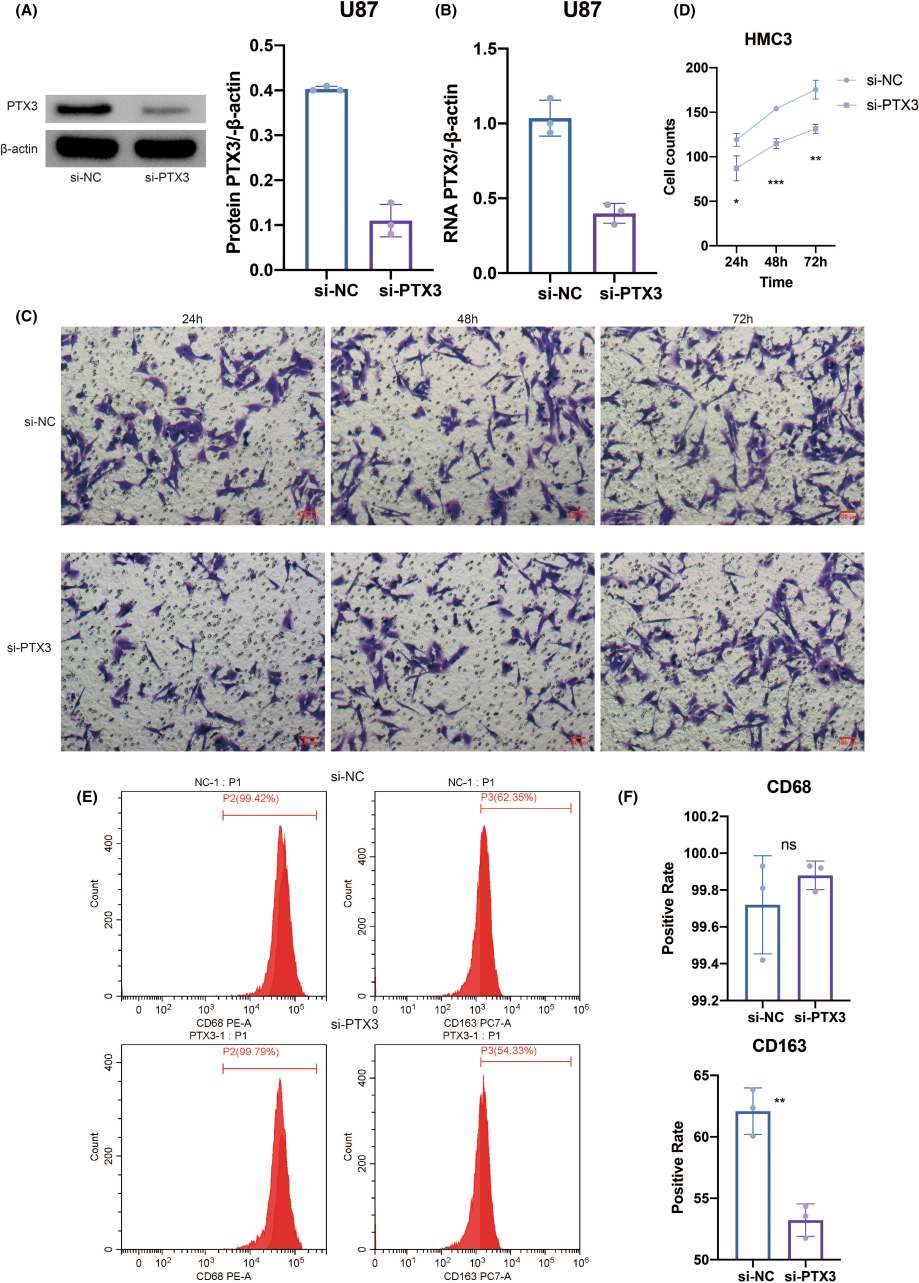
U87‐derived PTX3 mediated the migration and polarization of HMC3. (A) Western blotting results of PTX3 expression in NC and siRNA groups. (B) qPCR results of PTX3 expression in NC and siRNA groups. (C) Representative images of transwell assay for migration of HMC3 in NC and siRNA groups in different time points. (D) Statistical analysis of transwell assay. (E) Flow cytometry assay results of CD68 and CD163 expression in NC and siRNA groups. (F) Statistical analysis of flow cytometry assay.

To further elucidate the regulatory role of PTX3 in macrophages, in vitro experiments of coculture between U251 cells and HMC3 cells (microglia) were performed as microglia shared similar biological functions with macrophages in the central nervous system. Western blotting results showed that the si‐PTX3 group had significantly decreased protein expression of PTX3 compared with the si‐NC group (Figure 7A). RT‐qPCR results also proved that the si‐PTX3 group had considerably reduced RNA expression of PTX3 compared with the si‐NC group (Figure 7B). The coculture system between HMC3 cells and U251 cells for transwell assay was visualized in Figure 3C. Representative images of HMC3 cells in the si‐PTX3 and the si‐NC groups were obtained after coculturing for 24, 48, and 72 h (Figure 7D). Notably, HMC3 cells had significantly decreased ability in migration in the si‐PTX3 group compared with the si‐NC group at each time point (Figure 7E). The coculture system between HMC3 cells and U251 cells for flow cytometry assay was visualized in Figure 3F. Flow cytometry assay showed that CD68 was more expressed in the si‐PTX3 group than in the si‐NC group, while CD163 was significantly less expressed in the si‐PTX3 group than in the si‐NC group (Figure 7G,H). Furthermore, in vitro experiments of coculture between U87 cells and HMC3 cells (microglia) were performed. Western blotting results showed that the si‐PTX3 group had significantly decreased protein expression of PTX3 compared with the si‐NC group (Figure 8A). RT‐qPCR results also proved that the si‐PTX3 group had considerably reduced RNA expression of PTX3 compared with the si‐NC group (Figure 8B). Representative images of HMC3 cells in the si‐PTX3 and the si‐NC groups were obtained after coculturing for 24, 48, and 72 h (Figure 8C). Notably, HMC3 cells had significantly decreased ability in migration in the si‐PTX3 group compared with the si‐NC group at each time point (Figure 8D). Flow cytometry assay showed that CD163 was significantly less expressed in the si‐PTX3 group than in the si‐NC group (Figure 8E and F). The above results suggested that PTX3 was potentially involved in the migration of microglia and tended to stimulate the inflammation‐resolving‐polarization of microglia.

### 3.10 PTX3 predicts immunotherapy response

Notably, PTX3 could significantly predict immunotherapy response in 10 murine immunotherapy cohorts (Figure S17A), in which responders were more likely to present high PTX3 expression. We also evaluated the biomarker relevance of PTX3 by comparing it with standardized biomarkers based on their predictive power of response outcomes and OS of human immunotherapy cohorts. Interestingly, PTX3 alone had an AUC of >0.5 in 10 of the 25 immunotherapy cohorts (Figure S17B). PTX3 exhibited a higher predictive value than TMB, T.Clonality, and B. Clonality, with AUC values of >0.5 in eight, nine, and seven immunotherapy cohorts. The predictive value of PTX3 was, however, lower than the MSI score (AUC >0.5 in 13 immunotherapy cohorts), CD274 (AUC >0.5 in 21 immunotherapy cohorts), TIDE (AUC >0.5 in 18 immunotherapy cohorts), IFNG (AUC >0.5 in 17 immunotherapy cohorts), and CD8 (AUC >0.5 in 18 immunotherapy cohorts).

## 4 DISCUSSION

Through systematic and comprehensive bioinformatics analysis and in vitro experiments, we revealed the immune infiltrating patterns of PTX3 in cancer based on large‐scale samples. We proved the regulatory role of PTX3 in the migration and polarization of macrophages in the tumor microenvironment of GBM. By its expression pattern in the TCGA cohort in our previous finding,^23^ there was a significant positive correlation between PTX3 expression and the grade of gliomas in the CGGA cohort. We found that PTX3 expression in GBM was significantly up‐regulated, especially in the more malignant CL and ME subtypes. In our previous findings, PTX3 in CL and ME subtypes was also higher than that in NE and PN subtypes in GBM samples, and the AUC value of PTX3 was 84.2% in GBM based on the TCGA RNA‐seq data.^23^ In this study, the ROC curves further confirmed that PTX3 with the AUC values of 92.1% and 95.0% in LGG and Pan‐glioma could be a sensitive marker of the CL and ME subtypes. IDH has been proved to play an essential role in the prognosis of glioma patients.^32^

One recent study has also shown that IDH mutation could suppress the immune response to gliomas and induce immune evasion.^33^ Our results indicated that PTX3 expression in patients with IDH wild‐type was higher than that in patients with IDH mutants, suggesting that PTX3 was associated with worse survival and could be involved in immune evasion. The co‐deletion of chromosomes 1p and 19q is also a common event in gliomas.^34^ Previous studies have shown that loss of heterozygosity of 1p19q is more common in low‐grade gliomas, which increases chemosensitivity and helps patients with a better clinical outcome.^35^ We found that PTX3 expression in patients without 1p/19q co‐deletion was increased, which also suggested that PTX3 predicted worse survival. The MGMT gene is a classic tumor suppressor gene, and the MGMT protein is a DNA repair enzyme. MGMT methylated glioma is more sensitive to alkylated drugs, enabling MGMT to be an emerging target for increasing the sensitivity of chemotherapy in glioma.^34^ We observed that the high expression of PTX3 was related to the unmethylated gliomas. To sum up, these findings indicated that the upregulated expression of PTX3 correlated with a more malignant phenotype of glioma.

PTX3 in PAN and PNZ was higher than in other regions. Some studies have demonstrated that various immune factors produced by cell necrosis, such as IL‐1α and TLR, could induce the higher expression of PTX3,^36^, ^37^ which is consistent with our findings. We also found that PTX3 displayed higher expression in secondary and recurrent gliomas than in primary gliomas. Combined with our previous result, the OS, PFI, and DSS of LGG, GBM, and pan‐glioma patients with high expression of PTX3 were significantly lower than that of LGG, GBM, pan‐glioma patients with low expression of PTX3, respectively.^23^ We next established a nomogram based on PTX3 expression level and other risk factors to predict the prognosis of patients. This nomogram appeared to effectively serve as a tool for the clinical management of glioma patients.

Additionally, we found that PTX3 recruited multiple immune infiltrating cells and stromal cells, including CD4+/CD8 + T cells, NK cells, macrophages, fibroblasts, endothelial cells, and so on. A high expression level of PTX3 may promote Treg differentiation and inhibit the proliferation of T cells, especially CD4 positive αβ T cells. In addition, increased expression of PTX3 was negatively correlated with B cell activation, proliferation, and expression level of IgG, which indicated the inhibited humoral immunity. Studies have shown that PTX3 may hinder the presentation of apoptotic cell‐derived antigens,^38^ which supports that PTX3 may inhibit the anti‐tumor immune process in the tumor microenvironment. But PTX3 was also found to promote Th1 differentiation and Th1 immune response. Taken together, PTX3 was assumed to play dual roles in enhancing and suppressing cell‐mediated cancer immunity.

Targeting the combined immune checkpoints to improve the efficacy of immunotherapy has become promising.^39^ In our research, PTX3 is highly correlated with PD‐1, PD‐L1, CD274, CD276, and HAVCR2, all of which can mediate immune escape of gliomas.^6^ For example, the checkpoint molecule CD274 can suppress the immune response against gliomas, and the expression of PD‐1 on T cells can bind to CD274 on gliomas, which can inhibit the activity of T cells and mediate immune escape.^40^ Given the remarkable efficacy of immunotherapy targeting PD‐1, PD‐L1, CD276, HAVCR2, and other immune checkpoint molecules,^41^, ^42^, ^43^ the combined PTX3 and these classic immune checkpoints are expected to be a promising therapeutic target. It should also be noted that PTX3 positively correlated with CD80, an essential costimulatory molecule in activating T cells, which further proved the dual roles of PTX3 in cancer immunity.^44^

In single‐cell sequencing analysis, neoplastic cells with high or low PTX3 expression within the microenvironment were identified. Based on the pseudotime trajectory reconstructed by monocle, GBM cells would gradually differentiate from cell state 1 with low expression of PTX3 into cell state 2 with high expression of PTX3. GO enrichment analysis proved that GBM cells at cell state 2 had enhanced tumorigenic activity. Moreover, PTX3 was directly found to participate in the tumorigenic and immunogenic processes of GBM based on the GSEA results. Moreover, high PTX3 expression indicated a malignant molecular phenotype in GBM. The signaling pathways involved in the cell communications between the GBM and innate immune cells differed regarding PTX3 expression levels. Twenty‐four signaling pathways were significantly different between high PTX3 expression and low PTX3 expression. Inhibition of the FASLG signaling pathway can prevent the invasion of glioma.^45^ IL6, IL17, TWEAK, and VEGF signaling pathways induce glioma cell proliferation and migration.^46^, ^47^, ^48^, ^49^ Likewise, the PERIOSTIN signaling pathway has been reported to play an important role in glioma invasion and resistance to angiogenesis.^50^ Correspondingly, these signaling pathways were more closely related to GBM cells with high expression of PTX3, supporting its tumorigenic role. MIF,^51^ PTN,^52^ and EPO^53^ are also involved in the proliferation and angiogenesis of human glioma. However, these three signaling pathways were more closely related to GBM cells with low expression of PTX3, indicating the potential anti‐cancer ability of PTX3 in GBM. Our finding was supported by the previous study that PTX3 may play dual (pro‐tumor and anti‐tumor) roles in cancer, depending on tumor type, cellular source, and tumor microenvironment.^54^ Among the multiple regulons involved in cellular biological functions and cellular interactions, the top‐ranked regulons in neoplastic cells with high PTX3 expression were also found to regulate several tumorigenic processes such as regulation of apoptotic signaling pathway, positive regulation of notch signaling pathway, AMPK signaling pathway, PI3K‐Akt signaling pathway, and p53 signaling pathway. Among the critical regulons in neoplastic cells with high PTX3 expression, NFKB2 was recently found to mediate melanoma progression.^55^ RELB could upregulate PD‐L1 and facilitate immune evasion of prostate cancer.^56^ RELB could also promote glioma cell invasion.^49^ KLF5 was reported to regulate GBM angiogenesis.^57^ These previous studies greatly support our findings.

Furthermore, PTX3 has previously been reported to correlate with enhanced macrophage infiltration.^15^, ^58^ In our study, PTX3 could potentially mediate the interaction between GBM cells and macrophages via the VEGF, VISFATIN, LT, FSH, IL17, and IL10 signaling pathways. The significant co‐expression of PTX3 and macrophages/microglia markers, CD68 and CD163, based on multiplex immunofluorescence staining in tumor tissues of GBM, astrocytoma, HNSCC, ureteral urothelial carcinoma, THCA, urothelial carcinoma of the renal pelvis, BLCA, CESC, UCEC, squamous cell carcinoma of the penis, ovarian papillary cystadenocarcinoma, serous ovarian adenocarcinoma, TGCT, PRAD, and some corresponding normal tissues proved the potential role of PTX3 in the infiltration of macrophages/microglia. It should be noted that early efforts have categorized microglia/macrophages into M1 or M2 phenotypes. However, M1/M2 polarities are currently known to be oversimplified, and multiple functionally specific microglia/macrophage subpopulations have been identified.^59^ M1 polarization of activated macrophages has been linked with pro‐inflammatory activities while M2 polarization of activated macrophages has been linked with anti‐inflammatory activities.^59^ Although not identical to peripheral macrophage polarization, microglial activation phenotypes have been described as “M1‐like” and “M2‐like” since they have similar pro‐inflammatory and anti‐inflammatory characteristics.^60^ So, inflammation‐resolving could be a more accurate term to describe the M2‐like polarization in microglia in the central nervous system.^59^, ^60^ In this study, PTX3 was further found to mediate the migration and inflammation‐resolving‐polarization of microglia in the tumor microenvironment of GBM, in which microglia had enhanced ability in migration and increased expression of CD163 under the stimulation of PTX3. Notably, the flow cytometry result showed that CD68 was more expressed in the si‐PTX3 group than in the si‐NC group, indicating that PTX3 could potentially suppress CD68 expression in microglia.

In summary, we revealed the characteristics of PTX3 in the tumor development and the tumor immune microenvironment of gliomas. Notably, PTX3 was found to mediate the infiltration, migration, and polarization of macrophage/microglia in GBM. Generally, the immunosuppressive property of PTX3 makes it a potential new therapeutic target and prognostic marker for the treatment of gliomas. Our study was expected to provide the background support for further in‐depth research about PTX3 in gliomas.

## AUTHOR CONTRIBUTIONS

HZ, QC, YW, YZ, TL, WZ, WW, NZ, ZL, and SF designed and drafted the manuscript; HZ, QC, YW, YZ, TL, NZ, ZD, and ZW wrote figure legends and revised the article; andQC, HZ, and ZD conducted the data analysis. All authors read and approved the final manuscript.

## FUNDING INFORMATION

Financial support was provided by the Hunan Provincial Health Committee Foundation of China (202204044869), National Natural Science Foundation of China (Nos. 82073893, 82172685, and 82102848), Hunan Provincial Natural Science Foundation of China (Nos. 2019JJ50963, 2022JJ20095, and 2018SK2101), Xiangya Hospital Central South University Postdoctoral Foundation.

## CONFLICT OF INTEREST

All authors declare that they have no competing interests.

## Supporting information


Table S1

Table S2

Figures S1–S17

**Appendix S1** Supplementary Information

## Data Availability

All data used in this work can be acquired from the Gene Expression Omnibus (GEO; https://www.ncbi.nlm.nih.gov/geo/), the Cancer Genome Atlas (TCGA) datasets (https://xenabrowser.net/), the Chinese Glioma Genome Atlas (CGGA) datasets (http://www.cgga.org.cn/).
