# Supplementary material for: PTX3 mediates the infiltration, migration, and inflammation‐resolving‐polarization of macrophages in glioblastoma
Source: CNS Neurosci Ther. 2022 Jul 20;28(11):1748–66. doi: 10.1111/cns.13913 (PMC9532932; doi:10.1111/cns.13913)
Supplement: Supplementary file 2 — Table S2 [file CNS-28-1748-s002.docx]

| Factor | CGGA RNA-seq set | | | | TCGA RNA-seq set | | | |
| --- | --- | --- | --- | --- | --- | --- | --- | --- |
|  | **Univariate** | | **Multivariate** | | **Univariate** | | **Multivariate** | |
|  | **HR(95%Cl)** | **Pvalue** | **HR(95%Cl)** | **Pvalue** | **HR(95%Cl)** | **Pvalue** | **HR(95%Cl)** | **Pvalue** |
| PTX3 | 1.244(1.205-1.285) | <0.001 | 1.080(1.040-1.120) | <0.001 | 1.542(1.549-1.630) | <0.001 | 1.150(1.060-1.260) | <0.001 |
| Age | 1.029(1.021-1.036) | <0.001 | 1.010(1.000-1.010) | 0.030 | 1.064(1.054-1.074) | <0.001 | 1.030(1.020-1.050) | <0.001 |
| Gender |  | | | | | | | |
| Male | 1.000(0.840-1.190) | 0.972 | 0.960(0.810-1.140) | 0.649 | 1.230(0.950-1.590) | 0.123 | 1.230(0.940-1.610) | 0.136 |
| Grade |  | | | | | | | |
| WHO III | 3.153(2.396-6.533-4.149-11.145) | <0.001 | 2.820(2.140-3.730) | <0.001 |  |  |  |  |
| WHO IV | 8.533(2.396-6.533-4.149-11.145) | <0.001 | 4.860(3.600-6.550) | <0.001 |  |  |  |  |
| G3 |  |  |  |  | 3.333(2.265-12.176-4.904-26.960) | <0.001 | 1.940(1.280-2.920) | 0.002 |
| G4 |  |  |  |  | 18.118(2.265-12.176-4.904-26.960) | <0.001 | 2.680(1.550-4.630) | <0.001 |
| IDH mutation status |  | | | | | | | |
| Wildtype | 3.068(2.571-3.660) | <0.001 | 1.180(0.940-1.480) | 0.149 | 9.12(6.894-12.064) | <0.001 | 2.000(1.270-3.160) | 0.003 |
| 1p19q |  | | | | | | | |
| Non-codel | 4.372(3.284-5.821) | <0.001 | 2.700(1.990-3.670) | <0.001 | 4.369(2.815-6.782) | <0.001 | 2.080(1.260-3.440) | 0.004 |
