# Supplementary material for: PTX3 mediates the infiltration, migration, and inflammation‐resolving‐polarization of macrophages in glioblastoma
Source: CNS Neurosci Ther. 2022 Jul 20;28(11):1748–66. doi: 10.1111/cns.13913 (PMC9532932; doi:10.1111/cns.13913)
Supplement: Supplementary file 3 — Figures S1–S17 [file CNS-28-1748-s004.docx]

**Supplementary Figures**

**
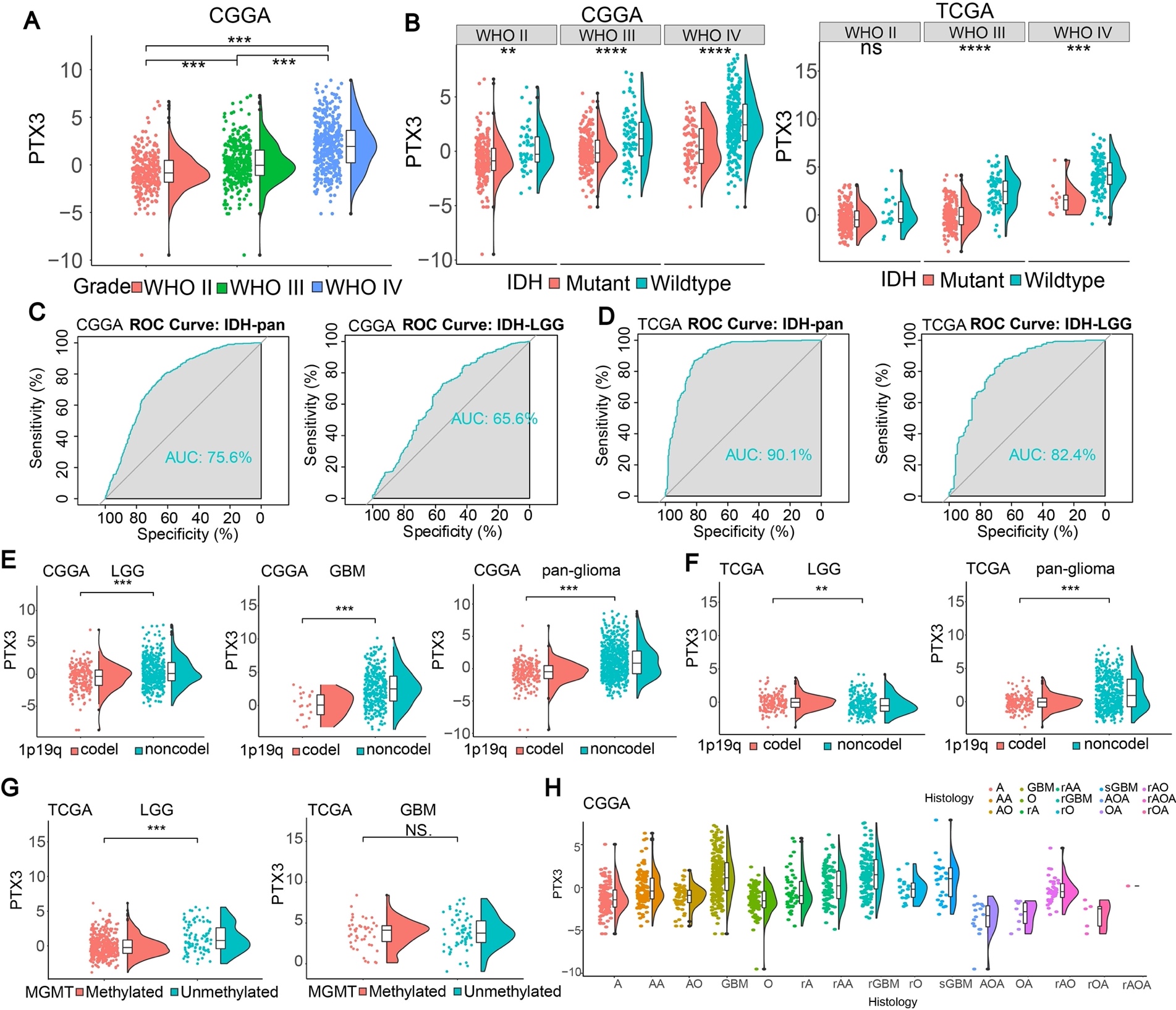
**

**Figure S1.** Inter-tumor and intra-tumor heterogeneous expression characteristics of PTX3 in gliomas. A. PTX3 expression in different grade of gliomas in CGGA dataset. B. PTX3 expression in IDH mutation and wildtype gliomas in CGGA and TCGA datasets. C. The ROC curve indicating the sensitivity and specificity of PTX3 expression in predicting IDH mutation in LGG and pan-glioma in CGGA dataset. D. The ROC curve indicating the sensitivity and specificity of PTX3 expression in predicting IDH mutation in LGG and pan-glioma in TCGA dataset. PTX3 expression in 1p19q codeletion and non-codeletion gliomas in E. CGGA dataset and F. TCGA dataset. G. PTX3 mRNA levels in MGMT methylated and unmethylated gliomas in TCGA dataset. H. PTX3 expression pattern with regard to histology of gliomas in CGGA dataset.


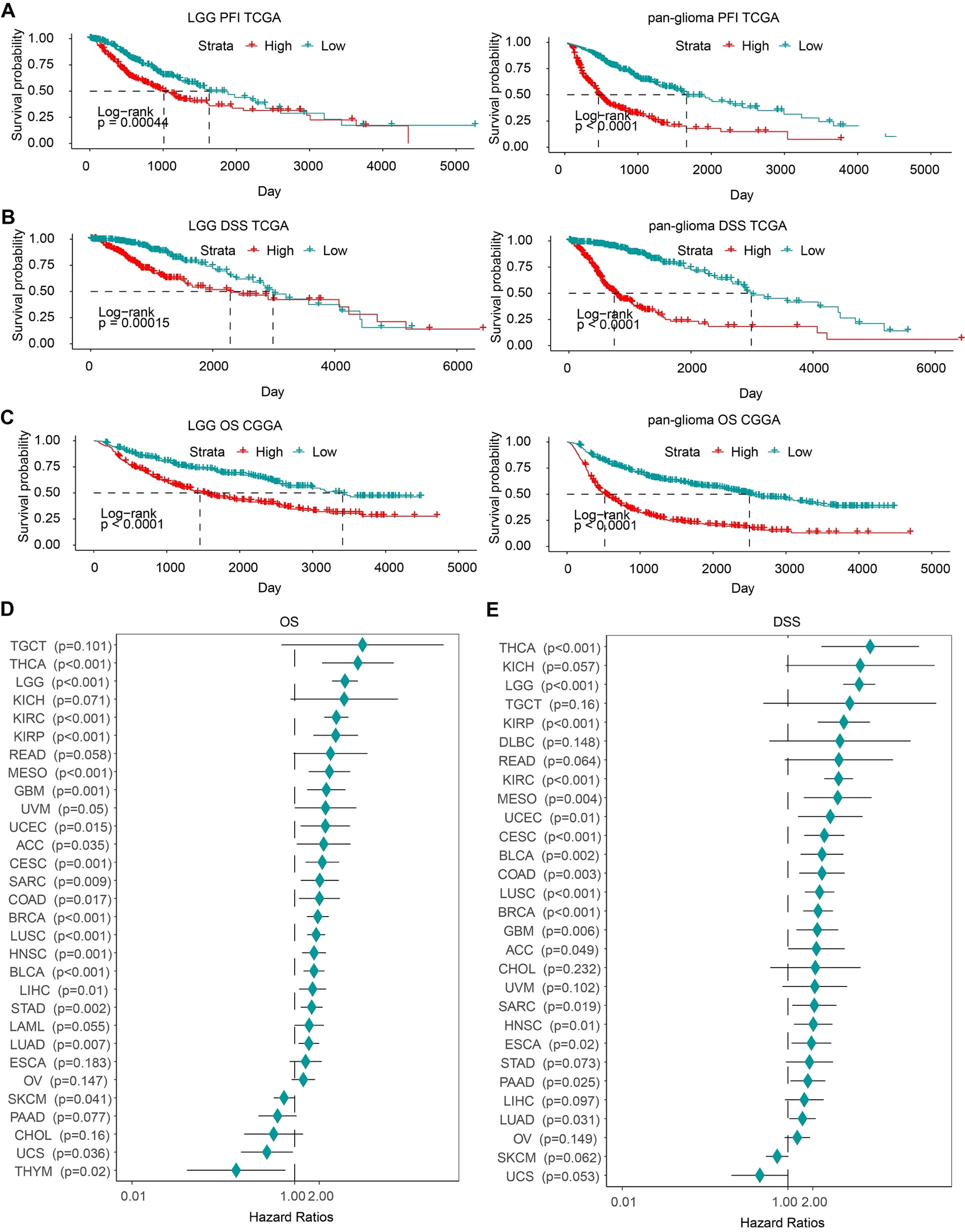


**Figure S2.** PTX3 expression is associated with poor survival in glioma patients. Kaplan-Meier curves of A. PFI and B. DSS based on high vs low expression of PTX3 in LGG and pan-glioma patients in TCGA dataset. C. Kaplan-Meier curves of OS based on high vs low expression of PTX3 in LGG and pan-glioma patients in CGGA dataset. P-values were obtained from the log-rank test. Forest plot depicting the univariate cox regression analysis of D. OS and E. DSS in patients from TCGA dataset based on PTX3 expression. ACC (Adrenocortical carcinoma), BLCA (Bladder urothelial carcinoma), BRCA (Breast invasive carcinoma), CESC (Cervical and endocervical cancers), CHOL (Cholangiocarcinoma), COAD (Colon adenocarcinoma), DLBC (Lymphoid Neoplasm Diffuse Large B-cell Lymphoma), ESCA (Esophageal carcinoma), GBM (Glioblastoma), HNSC (Head and Neck squamous cell carcinoma), KICH (Kidney Chromophobe), KIRC (Kidney renal clear cell carcinoma), KIRP (Kidney renal papillary cell carcinoma), LAML (Acute Myeloid Leukemia), LGG (Lower Grade Glioma), LIHC (Liver hepatocellular carcinoma), LUAD (Lung adenocarcinoma), LUSC (Lung squamous cell carcinoma), MESO (Mesothelioma), OV (Ovarian serous cystadenocarcinoma), PAAD (Pancreatic adenocarcinoma), READ (Rectum adenocarcinoma), SARC (Sarcoma), SKCM (Skin Cutaneous Melanoma) , STAD (Stomach adenocarcinoma), TGCT (Testicular germ cell tumors), THCA (Thyroid carcinoma), THYM (Thymoma), UCEC (Uterine Corpus Endometrial Carcinoma), UCS (Uterine Carcinosarcoma), UVM (Uveal Melanoma).

**
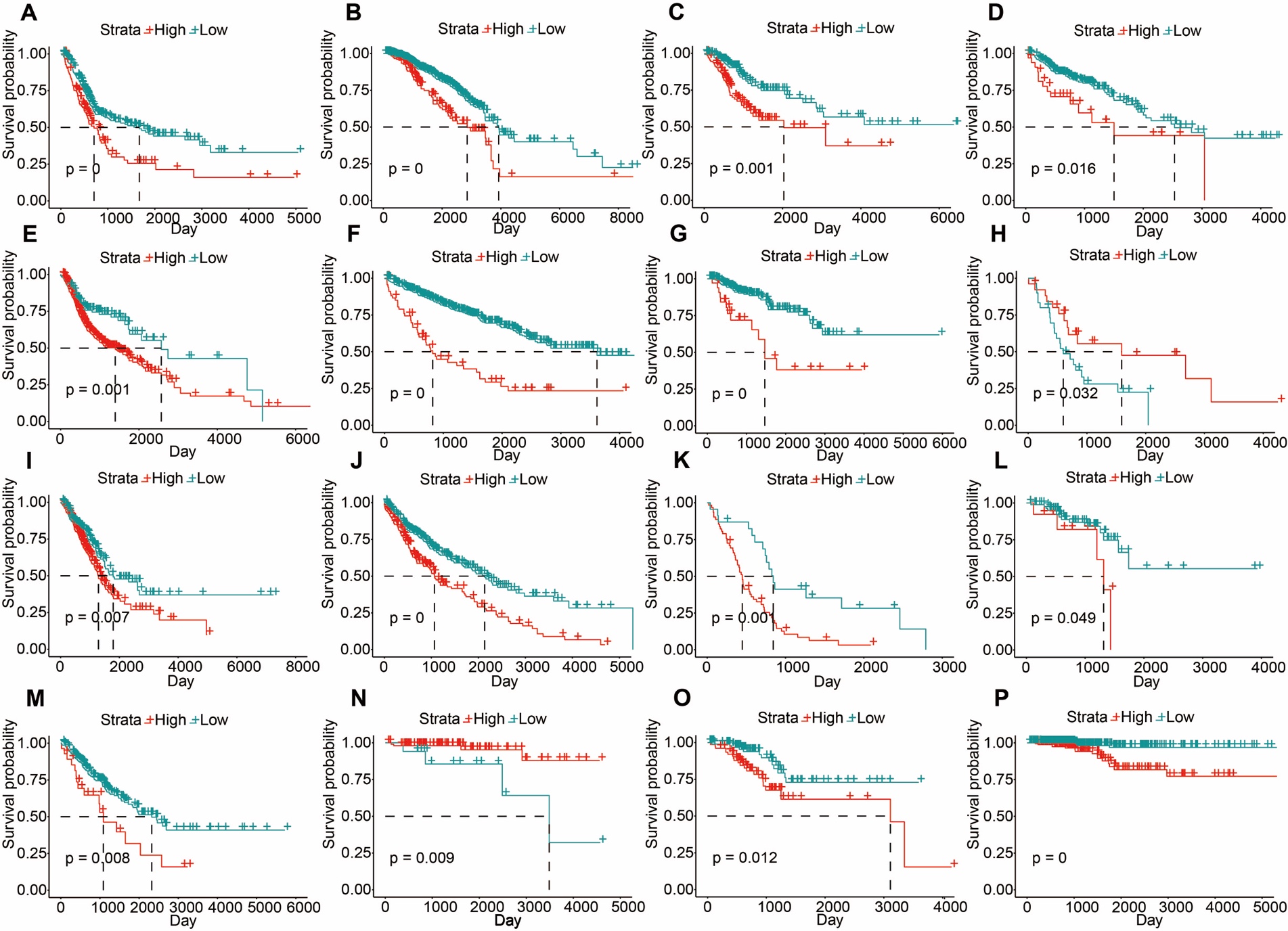
**

**Figure S3.** Kaplan-Meier analysis using high vs low PTX3 expression for OS in different cancer. A. BLCA, B. BRCA, C. CESC, D. COAD, E. HNSC, F. KIRC, G. KIRP, H. UCS, Uterine Carcinosarcoma; I. LUAD, J. LUSC, K. MESO, L. READ, M. SARC, N. THYM, O. UCEC, P. THCA. P-values were obtained from the log-rank test.

**
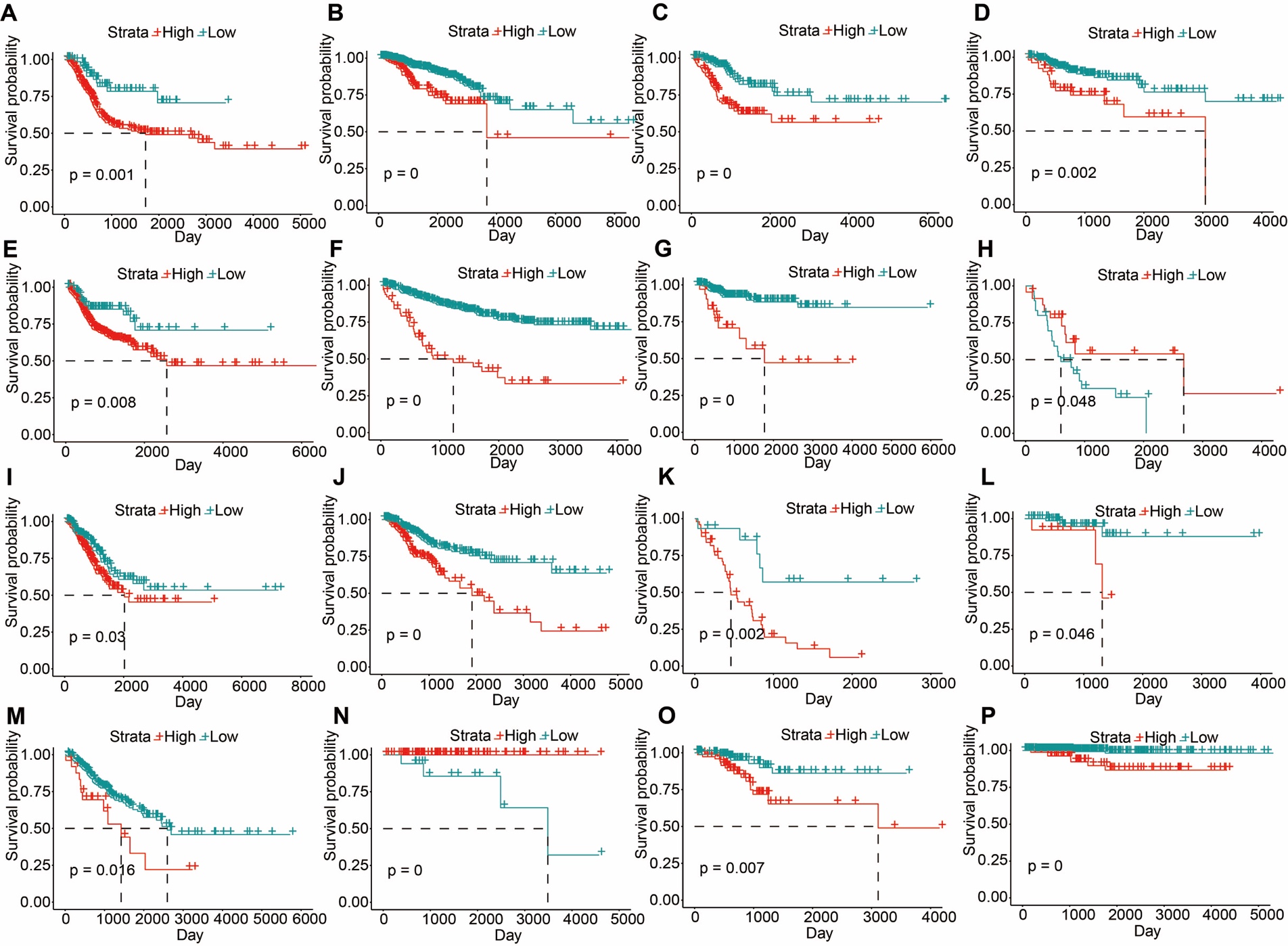
**

**Figure S4.** Kaplan-Meier analysis using high vs low PTX3 expression for DSS in different cancer. A. BLCA, B. BRCA, C. CESC, D. COAD, E. HNSC, F. KIRC, G. KIRP, H. UCS, Uterine Carcinosarcoma; I. LUAD, J. LUSC, K. MESO, L. READ, M. SARC, N. THYM, O. UCEC, P. THCA. P-values were obtained from the log-rank test. P-values were obtained from the log-rank test.

**
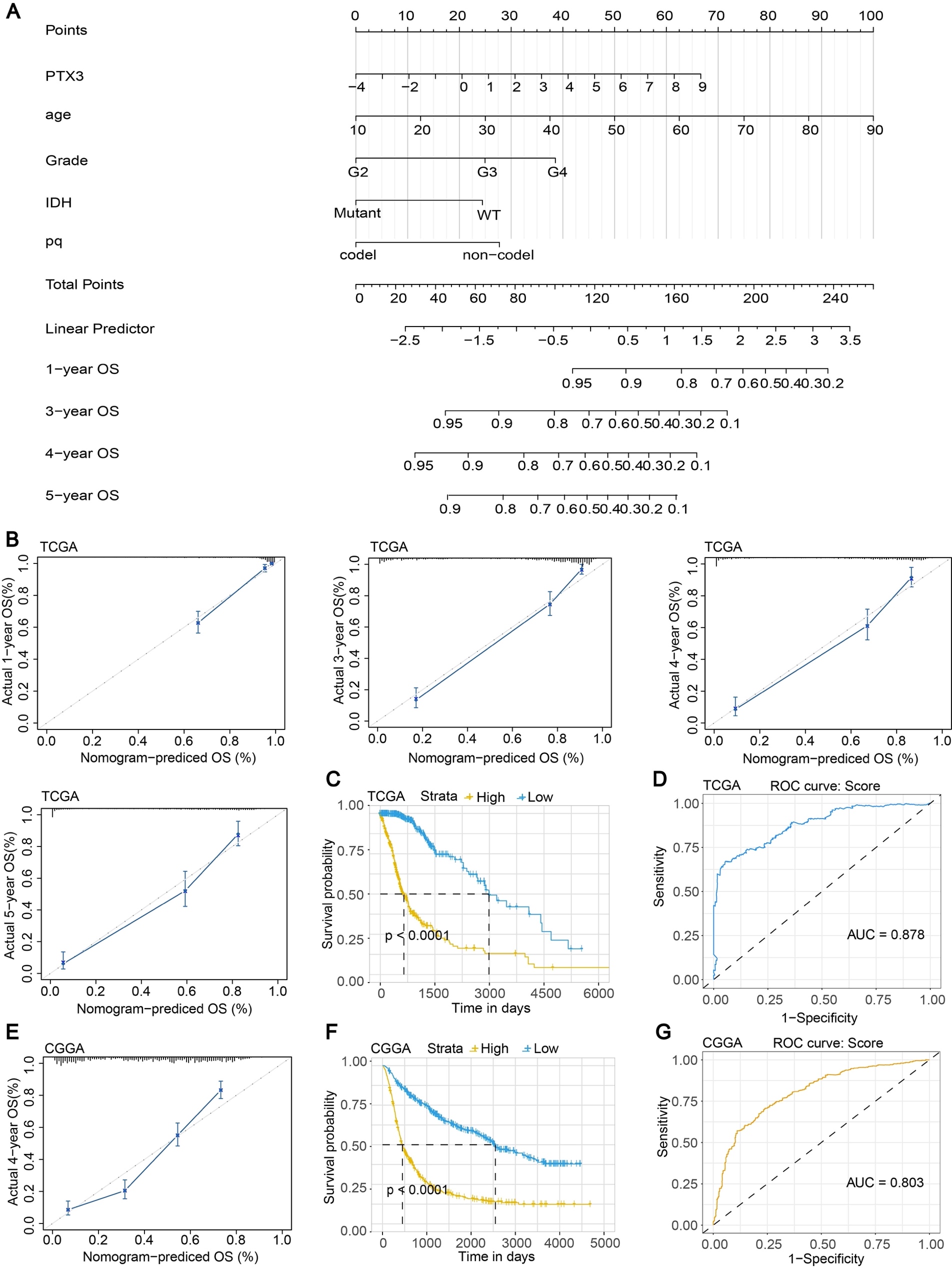
**

**Figure S5** The nomogram based on PTX3. A. Nomogram for predicting the clinical outcome of glioma patients with OS. Comparison between the nomogram-predicted OS and actual 1-year, 3-year,4-year,5-year OS in B. TCGA and E. actual 4-year in CGGA datasets. Kaplan-Meier analysis of OS based on high vs low expression of PTX3 in C. TCGA and F. CGGA datasets. The ROC curve to assess the sensitivity and specificity of the nomogram-derived score as a diagnostic biomarker in gliomas (D. AUC>=0.878 in TCGA dataset, G. AUC>=0.803 in CGGA dataset).


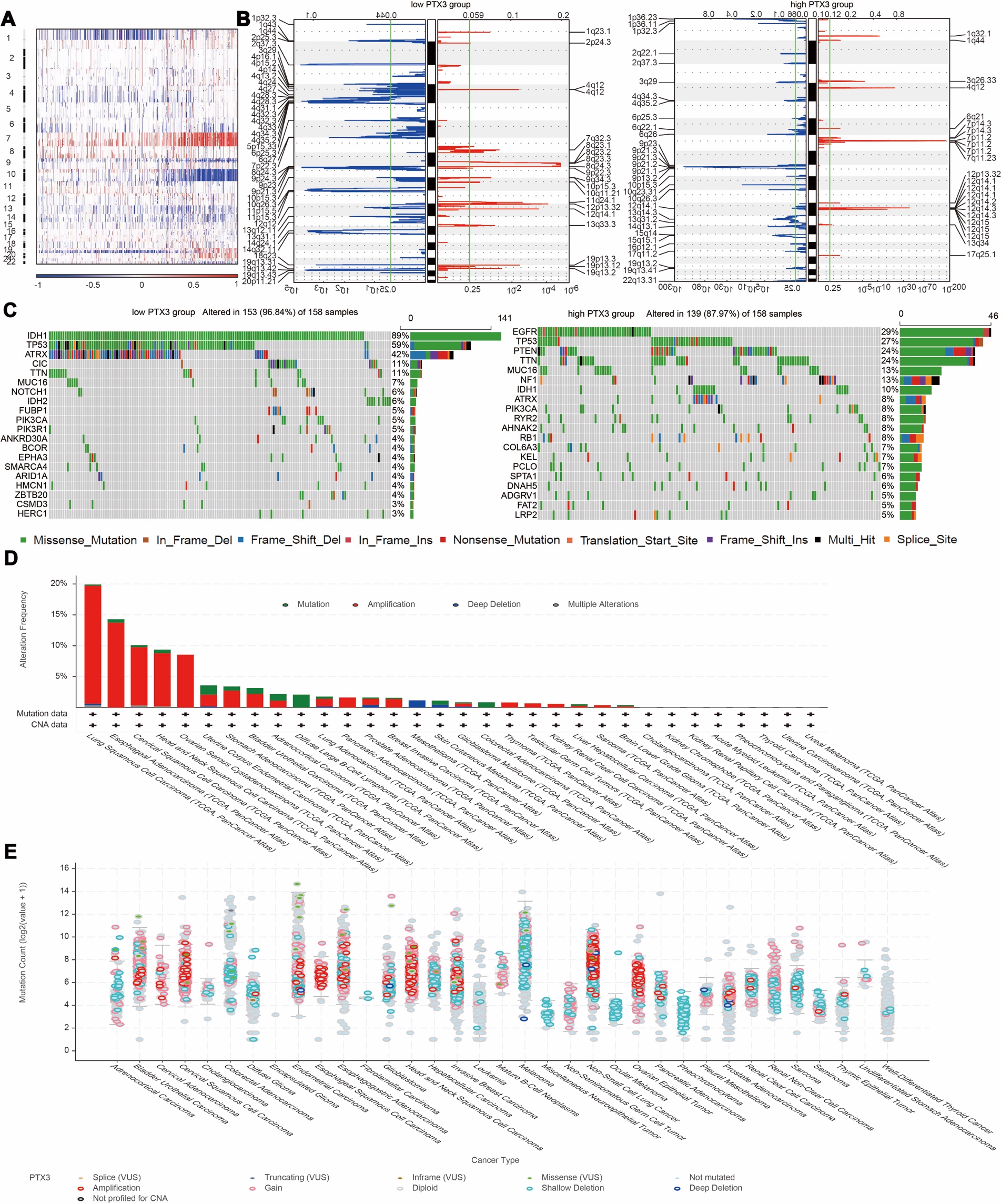


**Figure S6** Specific genomic profiles related to PTX3 expression. A. The overall copy number variation (CNV) according to the level of PTX3 expression. B. GISTIC 2.0 amplifications and deletions in gliomas with high and low PTX3 expression. The X axis represents the frequency of chromosome deletion (blue) or amplification (red). C. Differential somatic mutations were detected in gliomas with high and low PTX3 expression. D. Genetic aberrations of PTX3 in different cancer using TCGA pan-cancer panel of cBioPortal. E. The relevance of different cancer and PTX3 mutation count.

**
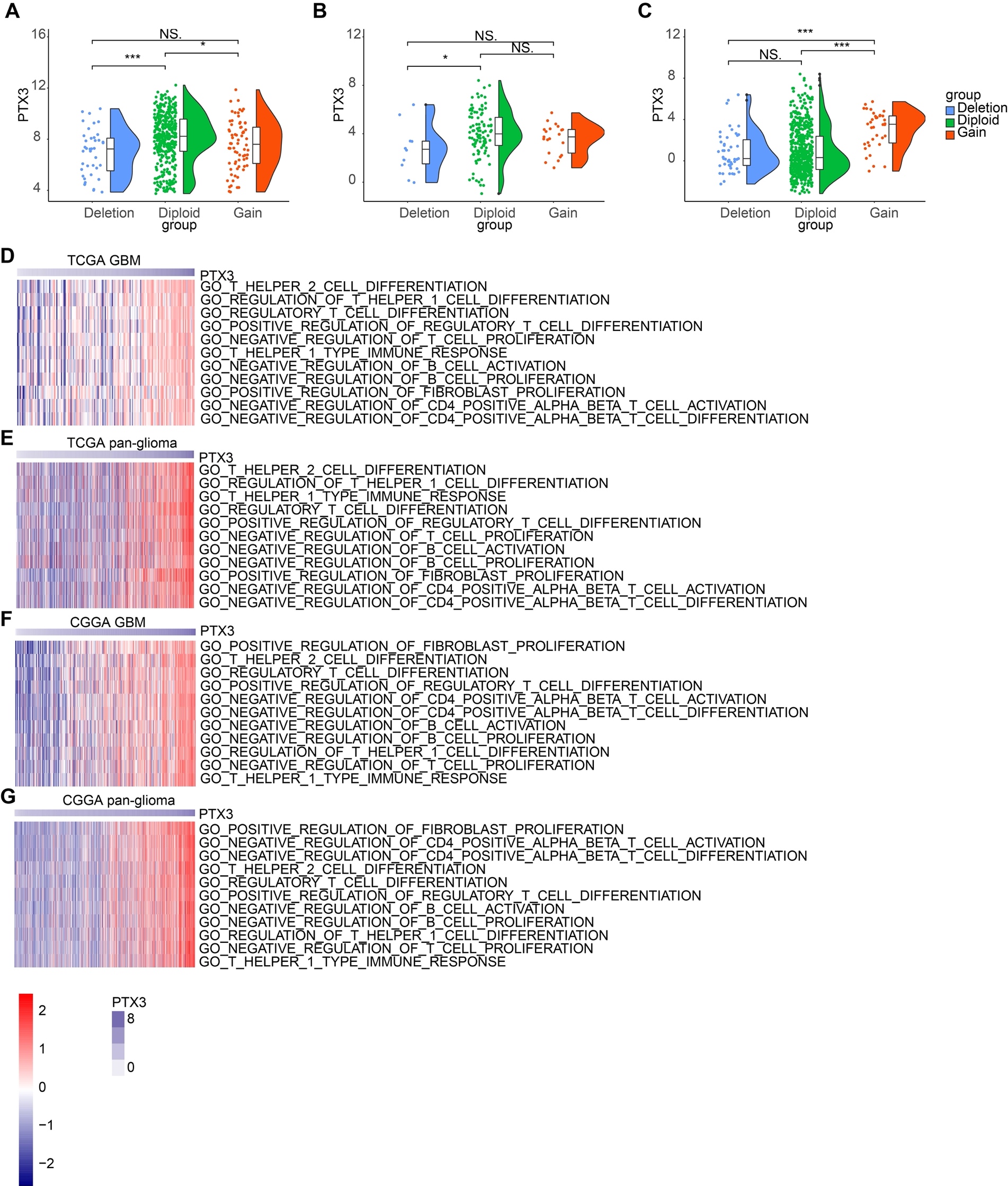
**

**Figure S7.** Connection between the PTX3 expression and PTX3 copy number (CN). A. Based on TCGA GBM array. B. Based on TCGA GBM seq C. Based on TCGA, LGG and GBM simples. PTX3 was closely related to immune functions in glioma. Gene ontology analysis showed that PTX3 was mostly involved in both cellular immunity and humoral immunity in D.GBM samples in TCGA dataset, E. pan-glioma samples in TCGA dataset and F. GBM samples in CGGA dataset, G. pan-glioma samples in CGGA dataset.

**
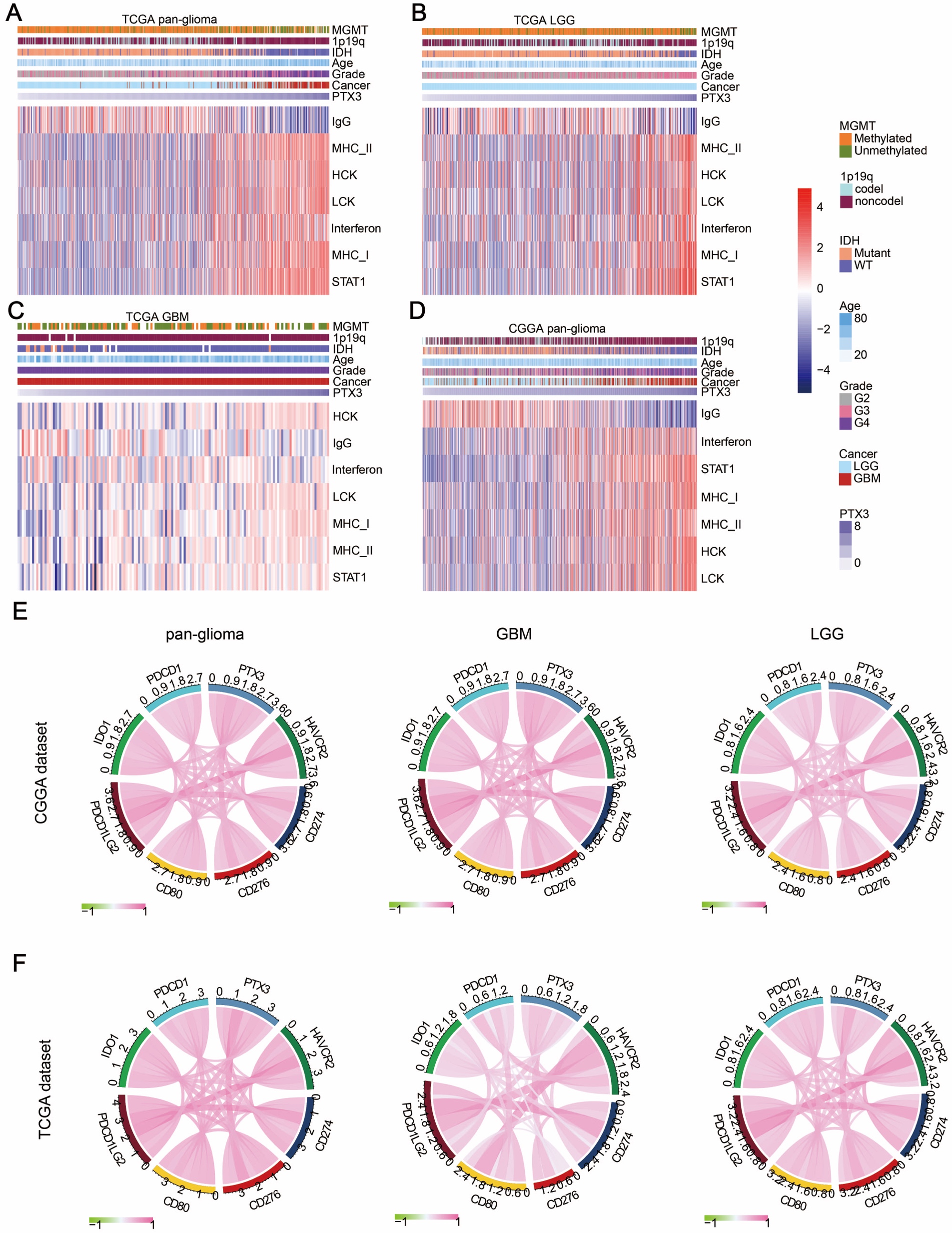
**

**Figure S8.** The correlation between PTX3 expression and inflammatory metagenes in A. pan-glioma samples in TCGA dataset, B. LGG samples in TCGA dataset, C. GBM samples in TCGA dataset, D. pan-glioma samples in CGGA dataset. Correlation of PTX3 and immune checkpoint molecules in pan-glioma samples, GBM samples and LGG samples in E. CGGA and F. TCGA datasets.

**
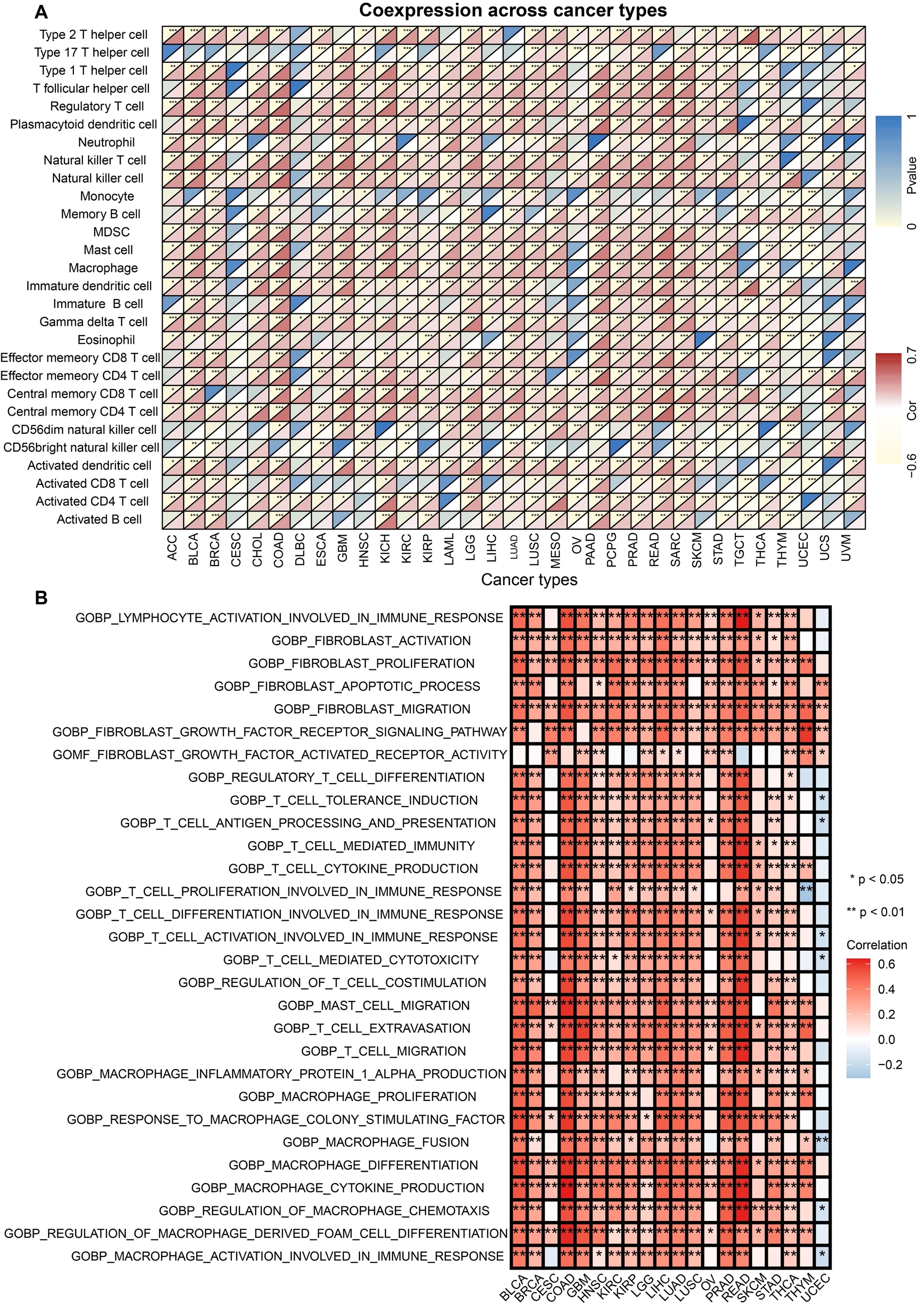
**

**Figure S9.** A. Correlation between immune cells and PTX3 in different cancer types including: ACC, BLCA, BRCA, CESC, CHOL, COAD, DLBC, ESCA, GBM, HNSC, KICH, KIRC, KIRP, LAML, LGG, LIHC, LUAD, LUSC, MESO, OV, PAAD, PCPG (Pheochromocytoma and Paraganglioma), PRAD (Prostate adenocarcinoma), READ, SARC, SKCM and STAD. B. Correlation between immune related biological pathways and PTX3 in different cancer types based on GO terms.


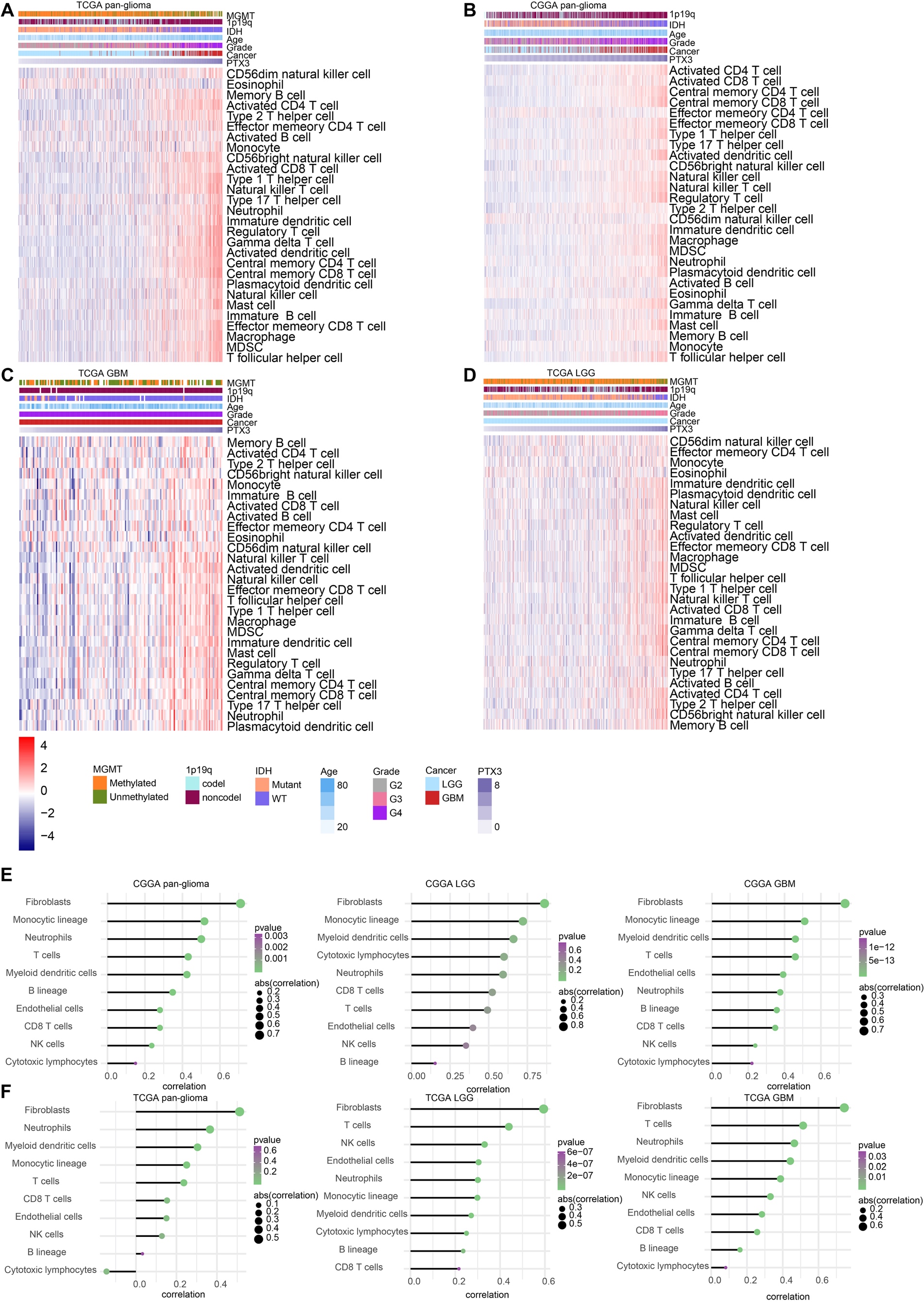


**Figure S10.** Correlation between PTX3 and 28 immune cell lineages in A. Pan-glioma samples in TCGA dataset, B. Pan-glioma samples in CGGA dataset, C. GBM samples in TCGA dataset, D. LGG samples in TCGA dataset. Red represents high expression and blue represents low expression, as shown on the scale. Correlation of PTX3 and 10 immune cell lineages in GBM (right), LGG (middle), pan-glioma(left) samples in E. CGGA and F. TCGA datasets.

**
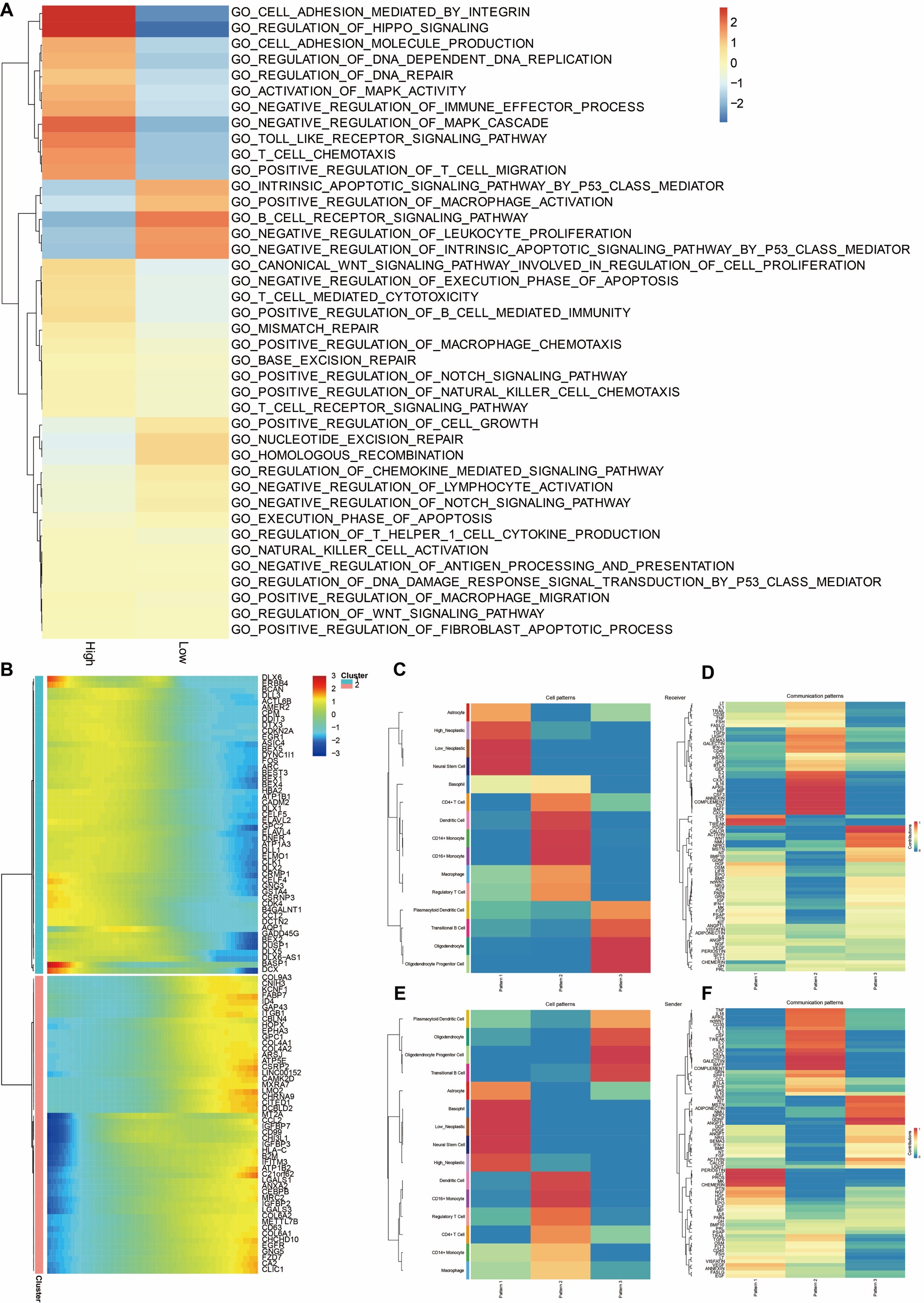
**

**Figure S11 Functional annotation and cellular communication roles of PTX3.** A. The heatmap for the relationship between biological functions of malignant cells and PTX3 expression. B. The heatmap for the trend of top 100 differentially expressed downregulated and upregulated genes with the increase in pseudotime. C. The heatmap of the distribution of 15 cell subtypes across the three receiver patterns. D. The heatmap for the three communication patterns of the receiver based on the gene expression. E. The heatmap of the distribution of 15 cell subtypes across the three sender patterns. F. The heatmap for the three communication patterns of the sender based on the gene expression.


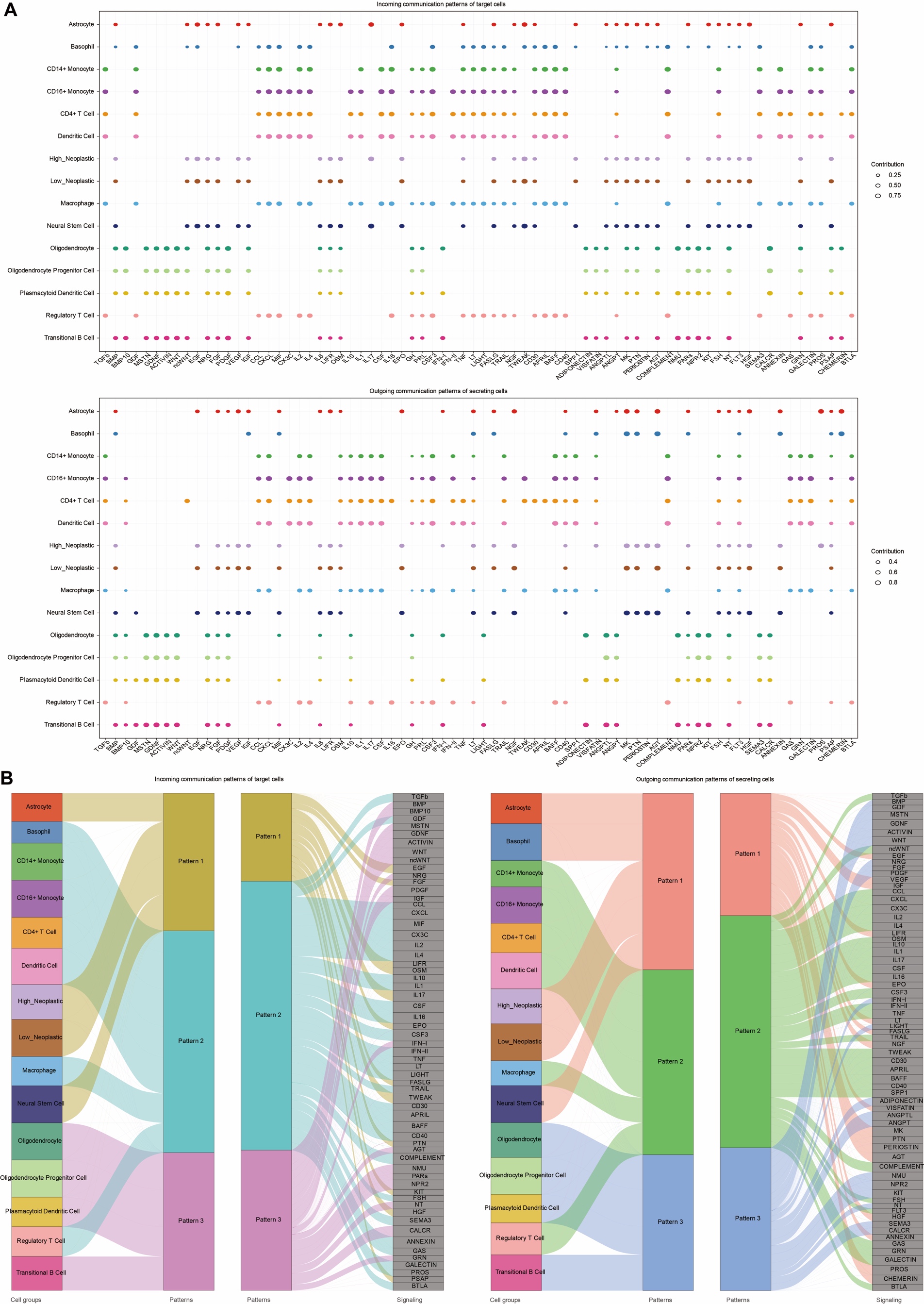


**Figure S12** Overall cellular communication patterns. A. Dot plot for the incoming and outcoming communication patterns of 15 cell types. B. Sankey plot for the incoming and outcoming communication patterns of 15 cell types.


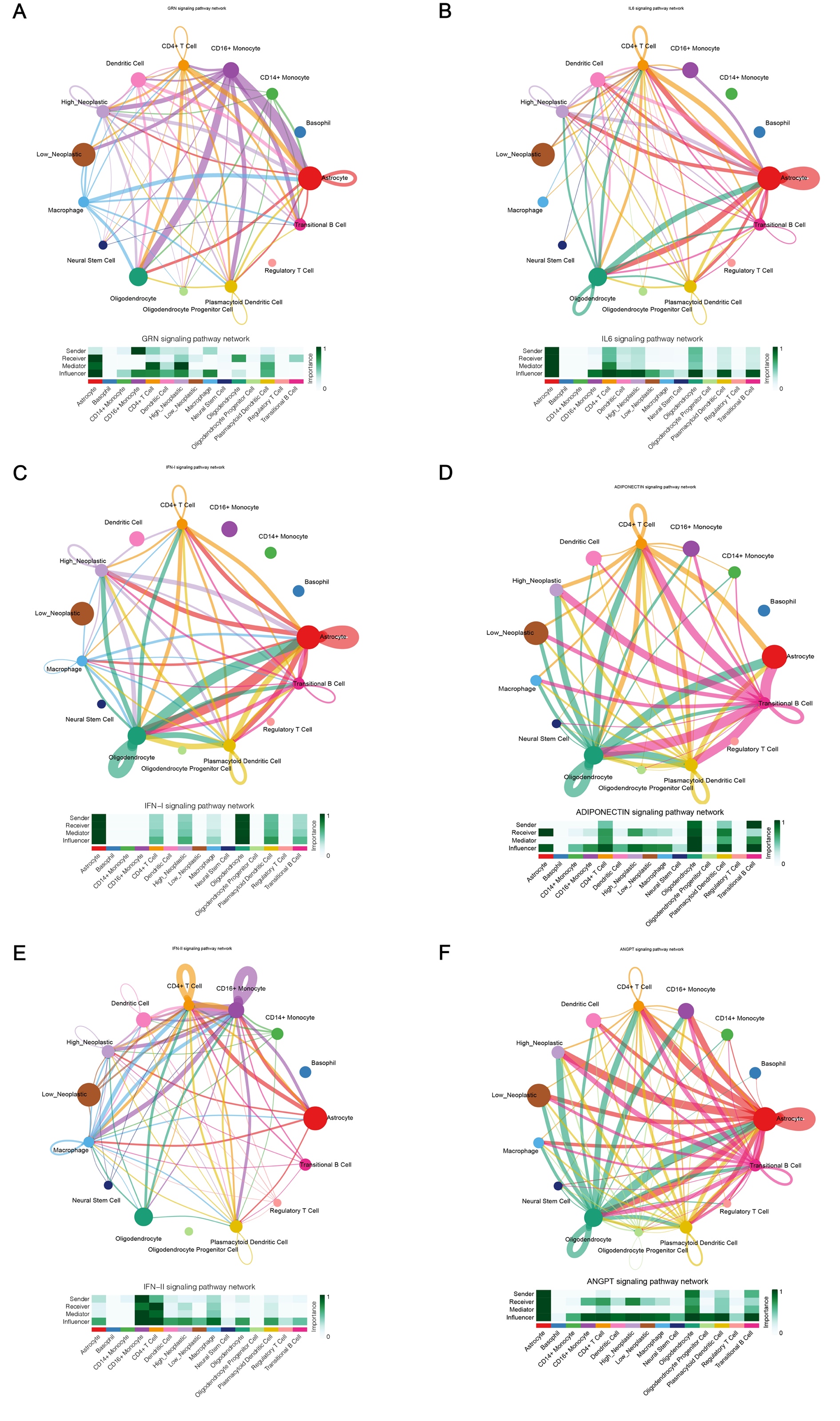


**Figure S13.** Cellular interaction within the two neoplastic cell clusters with different PTX3 expressions. The cellular interaction network for identified cell clusters in different signaling pathways including A. GRN, B. IL6, C. IFN-Ⅰ, D. ADIPONECTIN, E. IFN-Ⅱ, and F. ANGPT signaling pathway.


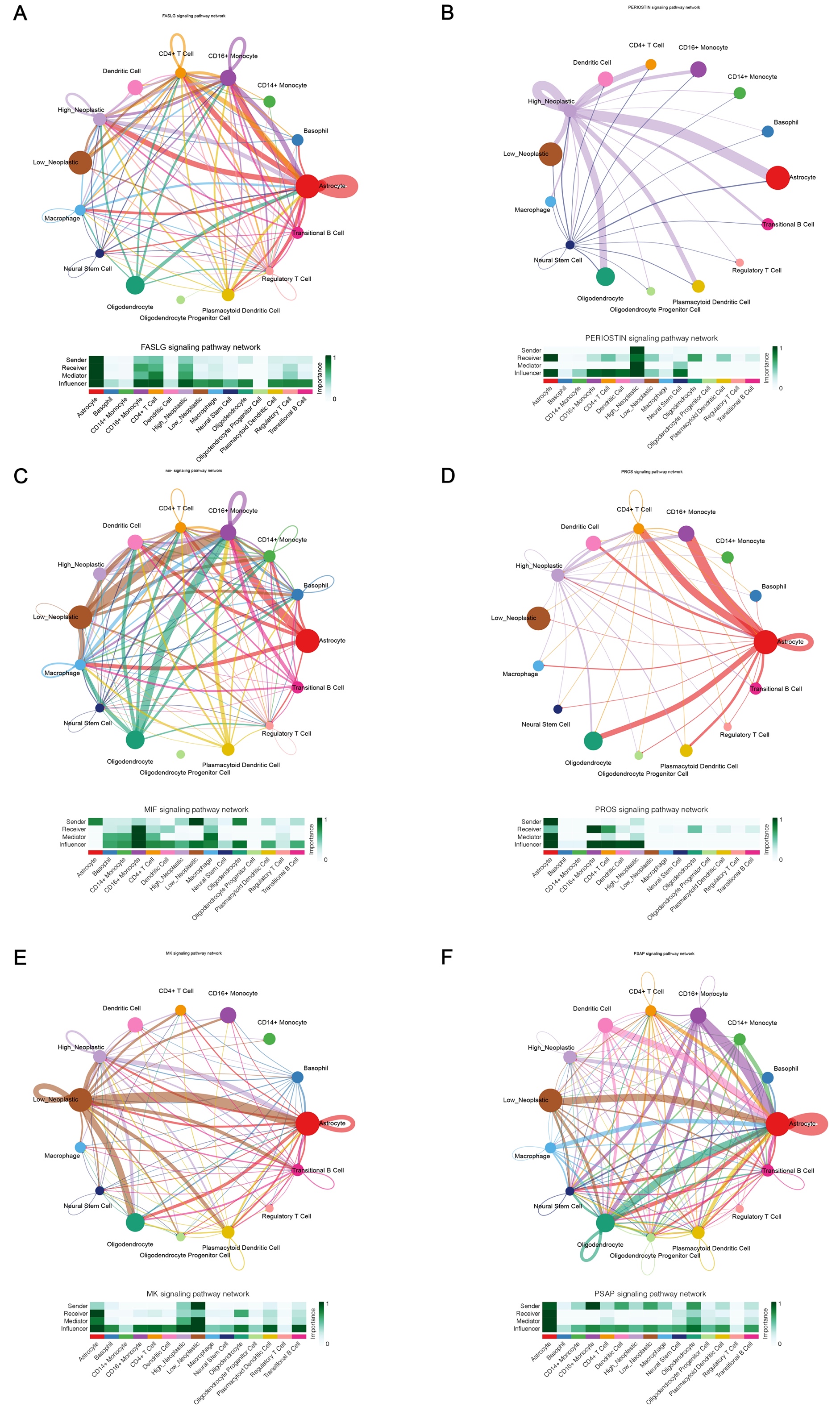


**Figure S14.** Cellular interaction within the two neoplastic cell clusters with different PTX3 expressions. The cellular interaction network for identified cell clusters in different signaling pathways including A. FASLG, B. PERIOSTIN, C. MIF, D. PROS, E. MK, and F. PSAP signaling pathway.


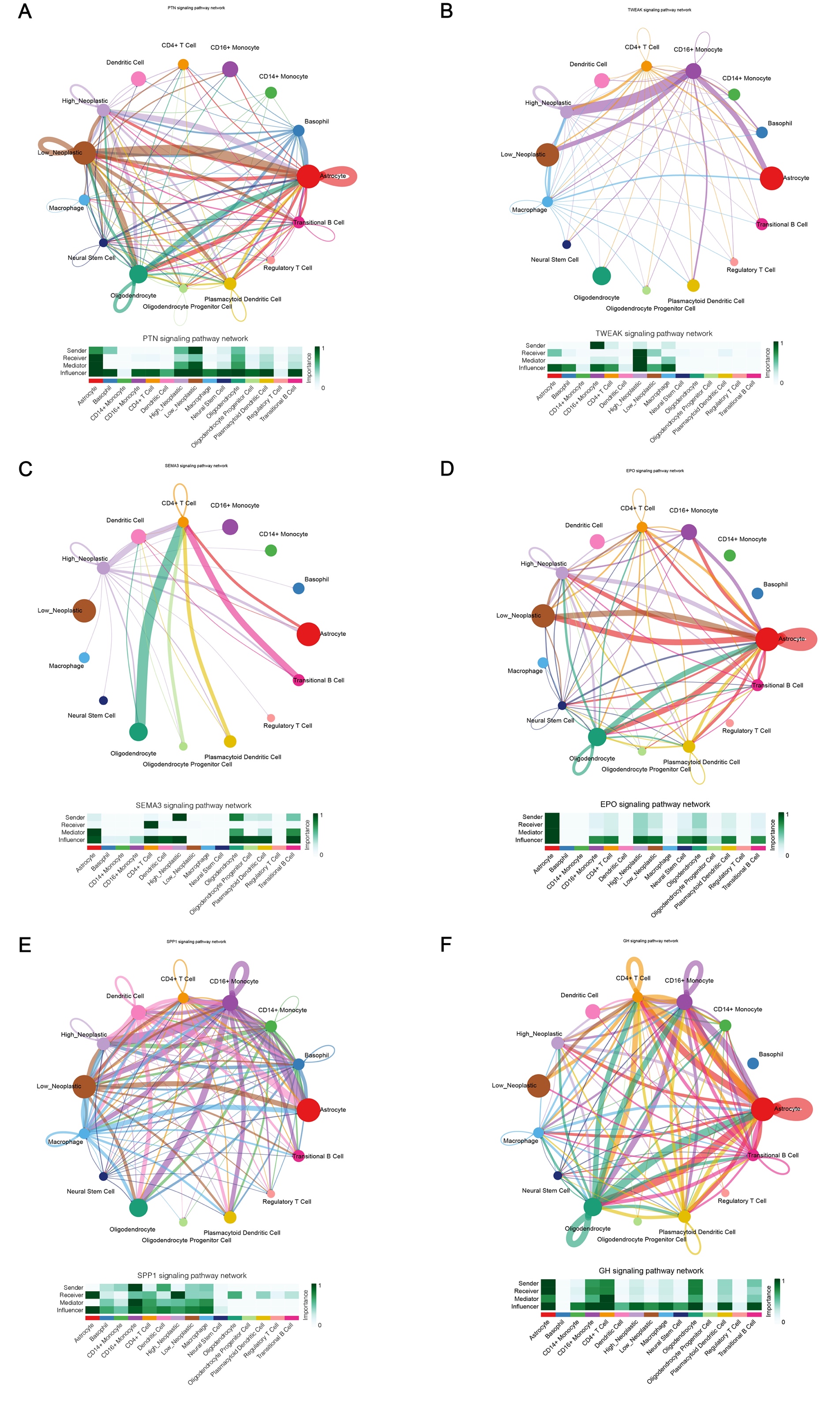


**Figure S15.** Cellular interaction within the two neoplastic cell clusters with different PTX3 expressions. The cellular interaction network for identified cell clusters in different signaling pathways including A. PTN, B. TWEAK, C. SEMA3, D. EPO, E. SPP1, and F. GH signaling pathway.


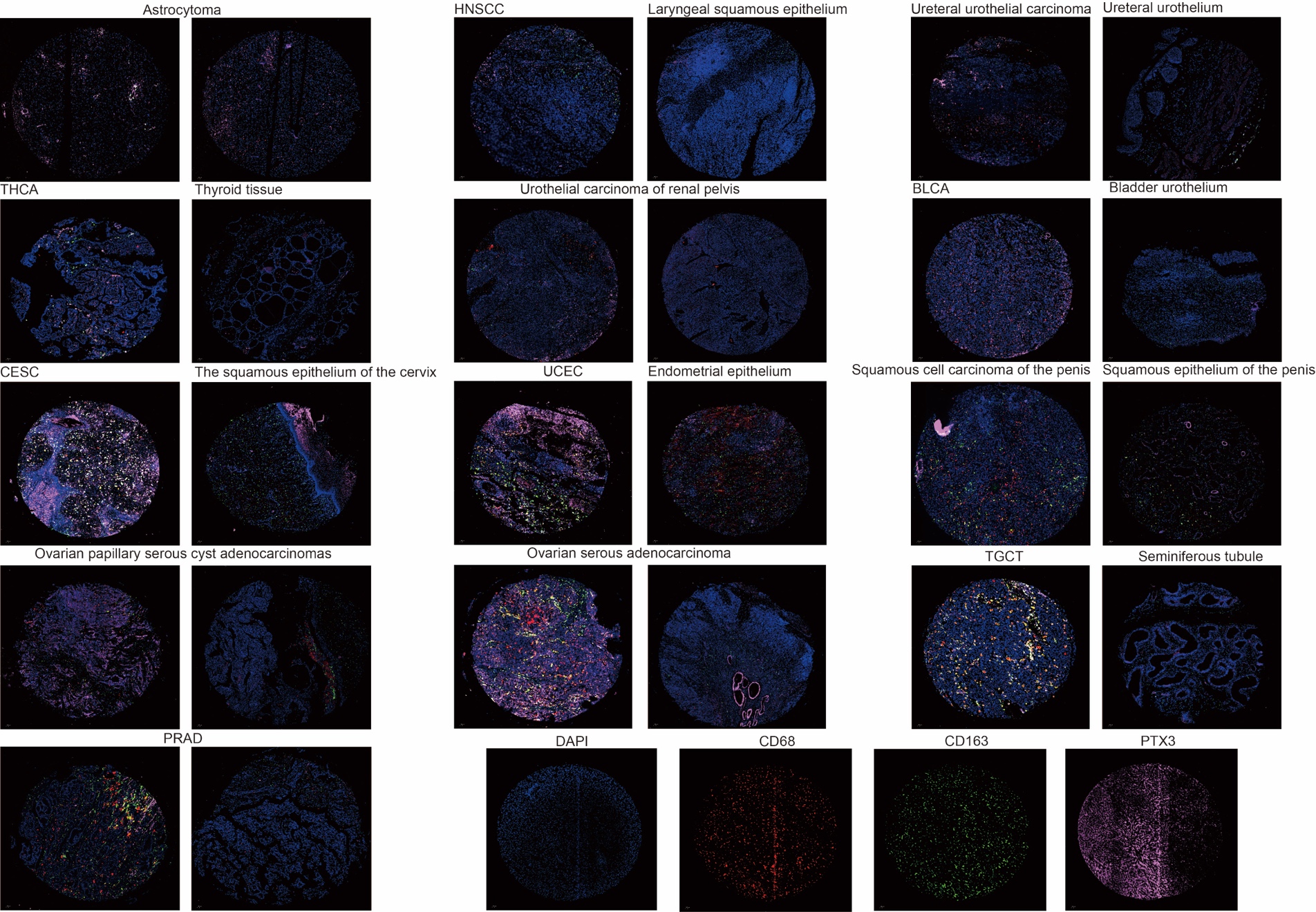


**Figure S16.** Multiplex immunofluorescence staining of CD68, CD163, PTX3, and DAPI. Multiplex immunofluorescence staining of CD68 (red), CD163 (green), PTX3 (pink), and DAPI (blue) in tumor tissues of astrocytoma, laryngeal squamous cell carcinoma (HNSCC), ureteral urothelial carcinoma, thyroid carcinoma (THCA), urothelial carcinoma of the renal pelvis, bladder carcinoma (BLCA), cervical squamous cell carcinoma and endocervical adenocarcinoma (CESC), uterine corpus endometrial carcinoma (UCEC), squamous cell carcinoma of the penis, ovarian papillary cystadenocarcinoma, serous ovarian adenocarcinoma, testicular germ cell tumors (TGCT), and prostate adenocarcinoma (PRAD) and some corresponding normal tissues (10X).


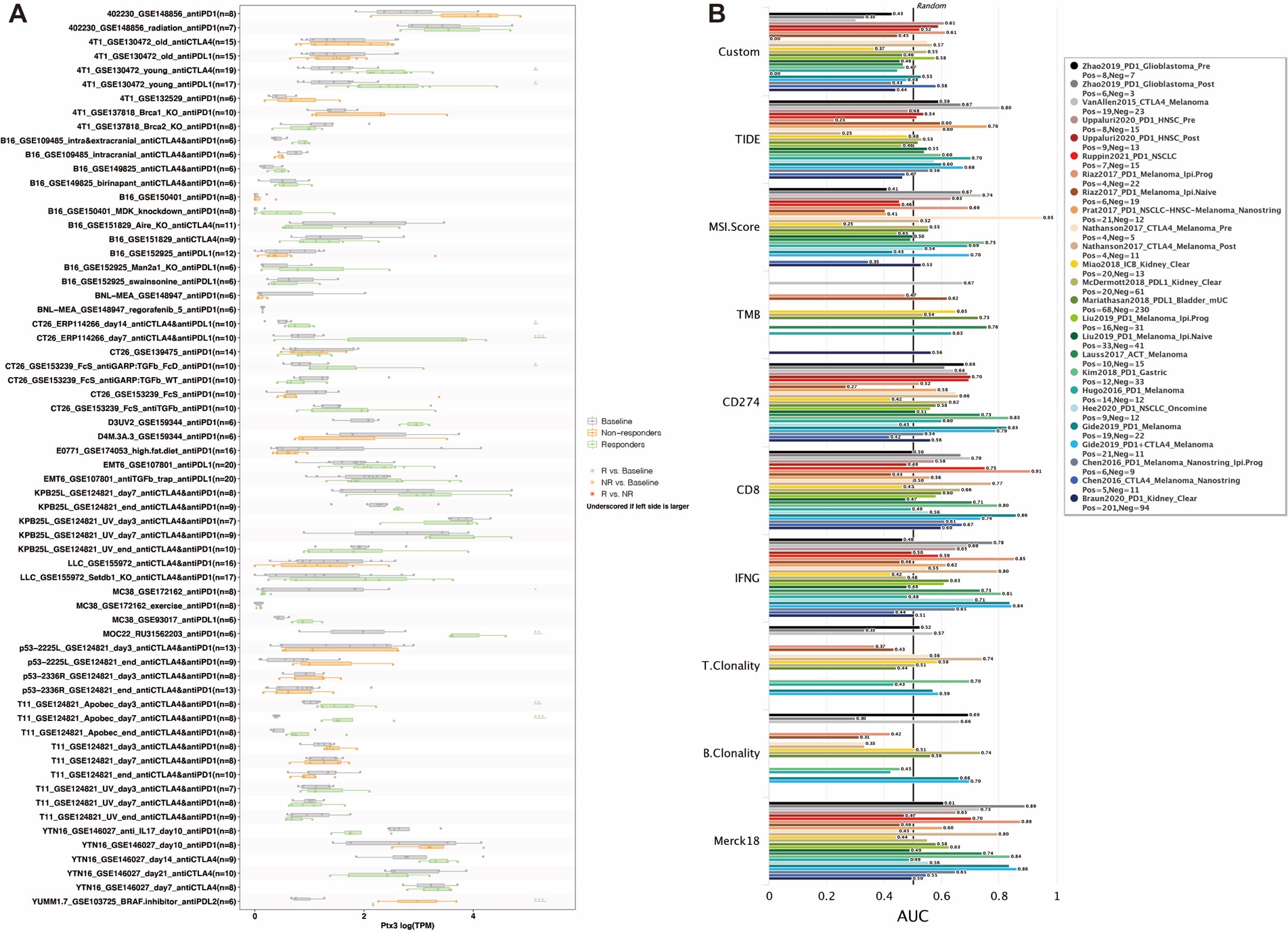


**Figure S17. The predictive value of PTX3 in immunotherapy response.** A. Box plot showing the predictive value of PTX3 in murine immunotherapy cohorts. B. Bar plot showing the biomarker relevance of PTX3 in human immunotherapy cohorts.

**Table S1.** Clinical characteristics of Xiangya cohort.

**Table S2.** Univariate and multivariate regression analyses for predicting overall survival in CGGA and TCGA datasets.
