# Supplementary material for: PTX3 mediates the infiltration, migration, and inflammation‐resolving‐polarization of macrophages in glioblastoma
Source: CNS Neurosci Ther. 2022 Jul 20;28(11):1748–66. doi: 10.1111/cns.13913 (PMC9532932; doi:10.1111/cns.13913)
Supplement: Supplementary file 4 — Appendix S1 Supplementary Information [file CNS-28-1748-s003.pdf]

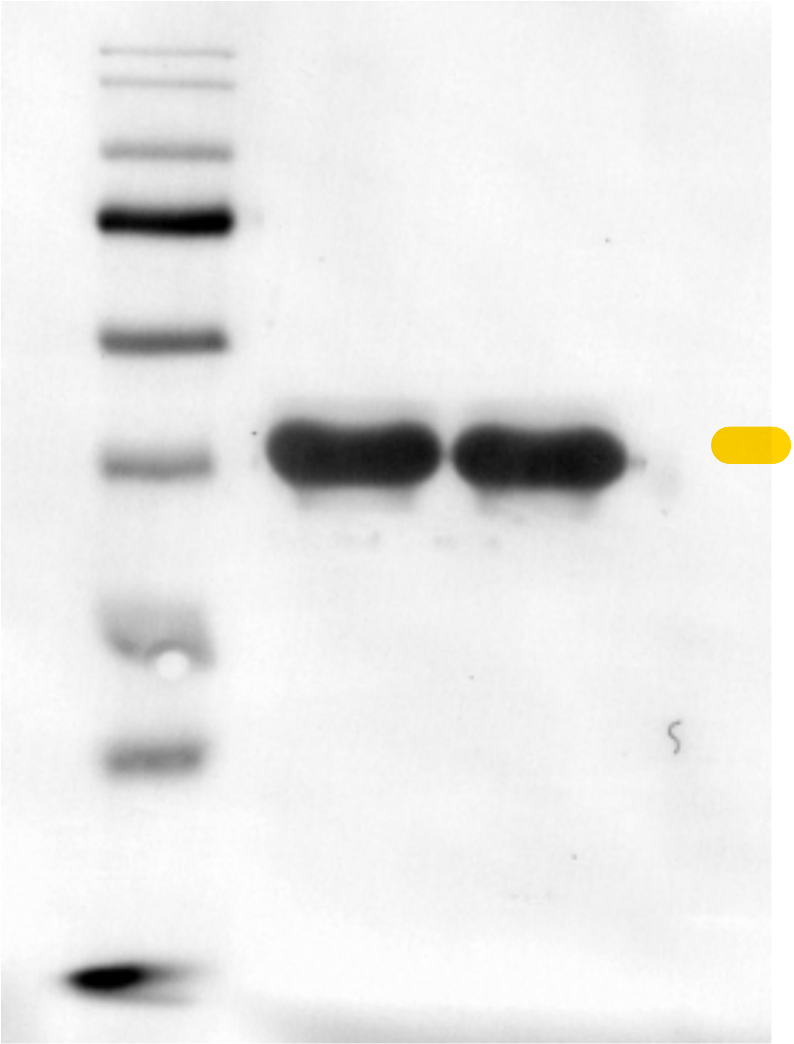

Full unedited gel/blot for Figure 7A: β-actin

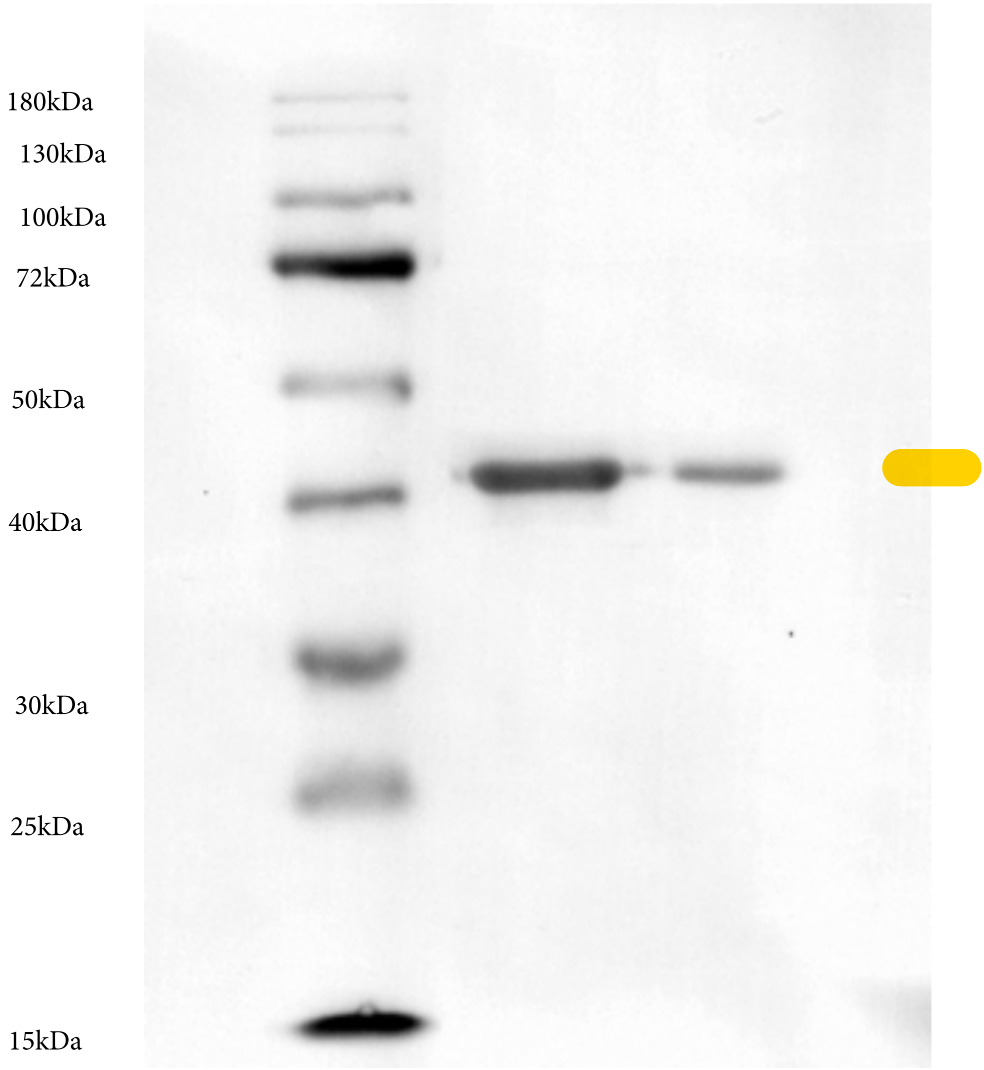

Full unedited gel/blot for Figure 7A: PTX3

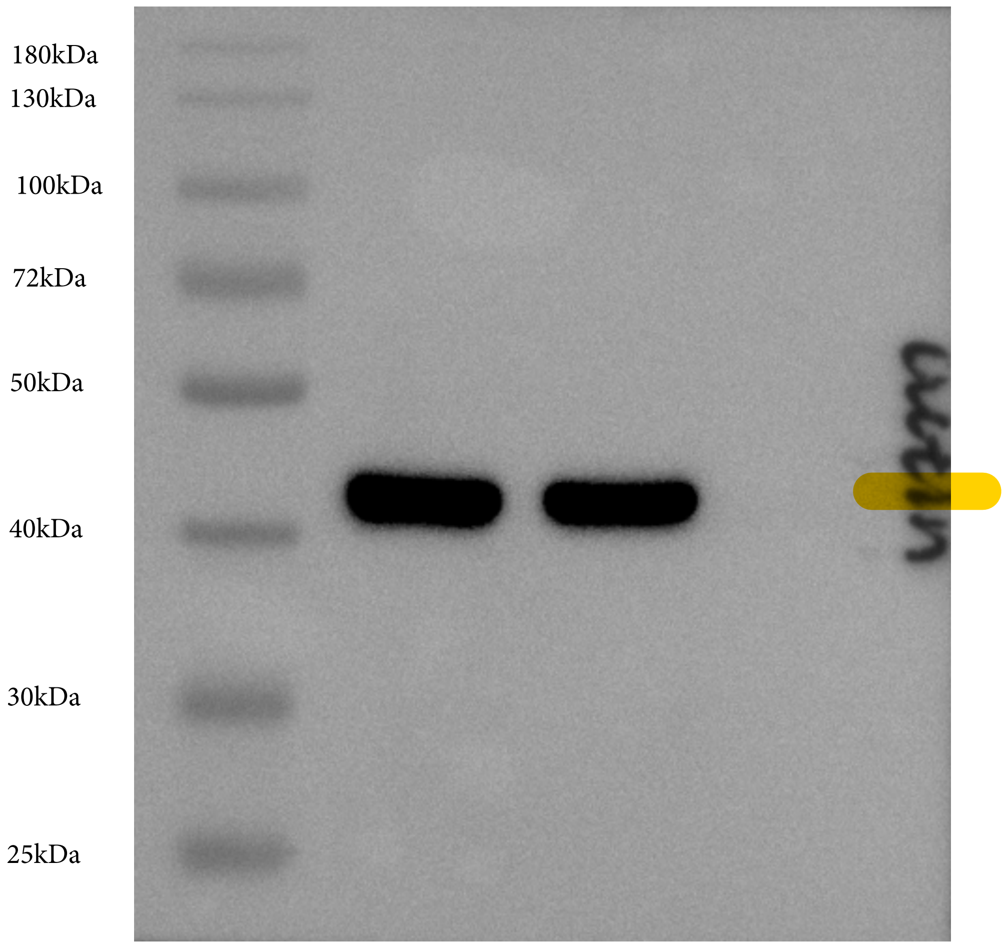

Full unedited gel/blot for Figure 8A: β-actin

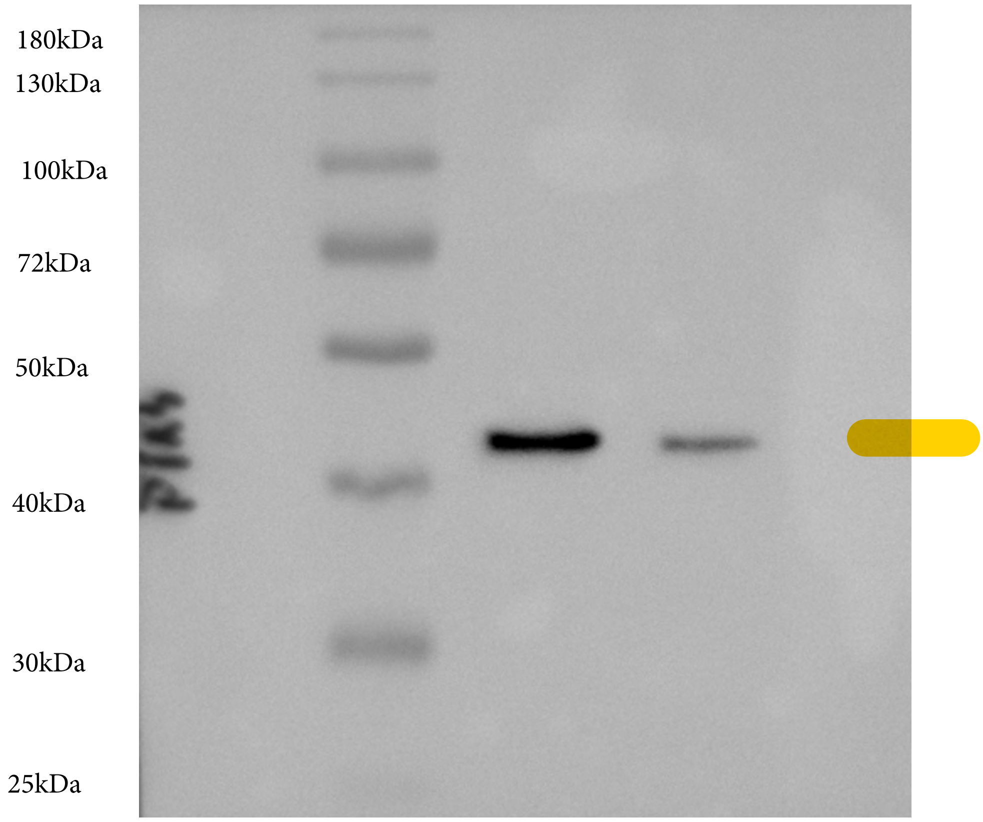

Full unedited gel/blot for Figure 8A: PTX3
